# DEF6(differentially exprehomolog) exacerbates pathological cardiac hypertrophy via RAC1

**DOI:** 10.1038/s41419-023-05948-0

**Published:** 2023-07-31

**Authors:** Yan Sun, Changlu Xu, Zhongxiu Jiang, Xi Jiang

**Affiliations:** 1 Department of Gastroenterology, Shengjing Hospital of China Medical University, 110022 Shenyang, Liaoning Province China; 2 Department of Cardiology, Shengjing Hospital of China Medical University, 110022 Shenyang, Liaoning Province China; 3 Department of Oncology, Shengjing Hospital of China Medical University, 110022 Shenyang, Liaoning Province China

**Keywords:** Genetics research, Heart failure

## Abstract

Pathological cardiac hypertrophy involves multiple regulators and several signal transduction pathways. Currently, the mechanisms of it are not well understood. Differentially expressed in FDCP 6 homolog (DEF6) was reported to participate in immunity, bone remodeling, and cancers. The effects of DEF6 on pathological cardiac hypertrophy, however, have not yet been fully characterized. We initially determined the expression profile of DEF6 and found that DEF6 was upregulated in hypertrophic hearts and cardiomyocytes. Our in vivo results revealed that DEF6 deficiency in mice alleviated transverse aortic constriction (TAC)-induced cardiac hypertrophy, fibrosis, dilation and dysfunction of left ventricle. Conversely, cardiomyocyte-specific DEF6-overexpression aggravated the hypertrophic phenotype in mice under chronic pressure overload. Similar to the animal experiments, the in vitro data showed that adenovirus-mediated knockdown of DEF6 remarkably inhibited phenylephrine (PE)-induced cardiomyocyte hypertrophy, whereas DEF6 overexpression exerted the opposite effects. Mechanistically, exploration of the signal pathways showed that the mitogen-activated extracellular signal-regulated kinase 1/2 (MEK1/2)-extracellular signal-regulated kinase 1/2 (ERK1/2) cascade might be involved in the prohypertrophic effect of DEF6. Coimmunoprecipitation and GST (glutathione S-transferase) pulldown analyses demonstrated that DEF6 can directly interact with small GTPase Ras-related C3 botulinum toxin substrate 1 (Rac1), and the Rac1 activity assay revealed that the activity of Rac1 is altered with DEF6 expression in TAC-cardiac hypertrophy and PE-triggered cardiomyocyte hypertrophy. In the end, western blot and rescue experiments using Rac1 inhibitor NSC23766 and the constitutively active mutant Rac1(G12V) verified the requirement of Rac1 and MEK1/2-ERK1/2 activation for DEF6-mediated pathological cardiac hypertrophy. Our study substantiates that DEF6 acts as a deleterious regulator of cardiac hypertrophy by activating the Rac1 and MEK1/2-ERK1/2 signaling pathways, and suggests that DEF6 may be a potential treatment target for heart failure.

## Introduction

Heart failure, the terminal stage of heart disease, limits the quality of life and imposes a major global health burden. Heart failure occurs since the heart is incapable of maintaining normal ventricular ejection to meet an individual’s needs even at normal filling pressures. Cardiac hypertrophy as a compensatory remodeling to pressure overload and other hypertrophic stimuli is a major risk factor of heart failure [1]. Hallmarks of pathological cardiac hypertrophy include an increment in cardiomyocyte size, excessive deposition of extracellular matrix, abnormal fetal gene expression and protein synthesis [1]. During the early stages, chronic pressure overload causes cardiac hypertrophy, which maintains or improve cardiac function by reducing stress on the ventricular wall. However, prolonged cardiac hypertrophy results in cardiac dysfunction, eventually promoting the progression of heart failure. Despite therapeutic advances in recent years, heart failure remains one of the most fatal conditions worldwide [2]. This relates in great part to poor knowledge about the underlying molecular mechanisms of pathological cardiac hypertrophy.

Differentially expressed in FDCP 6 homolog (Def6), which contains 631 amino acids, is mapped to human chromosome 6p21.31 [3]. Structurally, DEF6 is arranged with four domains, a Dbl homology-like domain (DHL), a pleckstrin homology domain (PH), an immunoreceptor tyrosine-based activation motif-like sequence, and an EF-hand motif from the C-terminus to the N-terminus [4, 5]. Because of its molecular structures and domains, Def6 was initially discovered as a Rho guanine nucleotide exchange factor (GEF) that regulates GTPases such as Rac1, RhoA, and Cdc42 [4, 6, 7]. However, DEF6 has a structure different from that of typical GEFs, in which the PH domain is located at the C-terminus of the DHL domain. The diversity of DEF6’s biological functions that are distinct from the typical GEFs may be ascribed to this atypical structure. Previous studies have indicated that DEF6 is expressed principally in the immune cells and participates in the regulation of adaptive immune responses and innate immunity. Among immune cells, Def6 exists mainly in T lymphocytes and plays pivotal roles in regulating development, activation, and function of them [7–11]. For instance, DEF6 can be phosphorylated by the tyrosine-protein kinases LCK21 and ITK22 and then activate small GTPases RHOA and Ras to promote NFAT1 activation, Ca^2+^ signaling, and T-cell adhesion [5, 10–12]. Many studies involving mice and humans have demonstrated that DEF6 deficiency is closely correlated with autoimmune diseases. Yi et al., Fanzo et al. and Chen et al. found that Def6-deficient mice exhibit inflammation and symptoms of autoimmune disorder as well as diverse immune defects, including abnormalities in T-cell expansion and TH cell differentiation, profound hypergammaglobulinemia, and autoantibody production [13–15]. DEF6 has also been reported to exist in myeloid cells, indicating that it regulates innate immunity [3, 16]. Additionally, Binder et al. found that DEF6 regulates bone remodeling by inhibiting inflammatory bone resorption and osteoclastogenesis [17]. Furthermore, DEF6 also involves in the tumorigenesis. It has been found to be overexpressed in renal cell carcinoma, oral squamous cell carcinoma, and ovarian cancer, and unfortunately, high expression levels of DEF6 indicate a poor prognosis [18–20]. Plenty of researches have verified the involvement of DEF6 in the immune system; however, no link has yet been established between DEF6 and cardiac hypertrophy. Numerous studies have supported the notion that many regulators of the immune system and cancers also participate in the regulation of cardiac hypertrophy [21–25], and considering that DEF6 is diffusely expressed in the heart [26], we sought to figure out whether DEF6 is implicated in cardiac hypertrophy.

We conducted a suite of experiments to explore this possibility. First, remarkably increased expression levels of DEF6 were observed in the hypertrophic hearts of mice processed with TAC and in cardiomyocytes administered with phenylephrine (PE). Then, DEF6 loss- and gain-of-function assays conducted both in vitro and in vivo revealed a deleterious regulatory role for DEF6 in pathological cardiac hypertrophy. In terms of mechanism, RAC1 and the MEK-ERK signaling cascade activation largely contributed to DEF6’s deleterious role in cardiac hypertrophy. In conclusion, activation of Rac1 and MEK-ERK signaling cascades are some of the mechanisms by which DEF6 accelerates the development of pathological cardiac hypertrophy.

## Results

### The expression of DEF6 is increased in hypertrophic hearts and cardiomyocytes

**Fig. 1:**
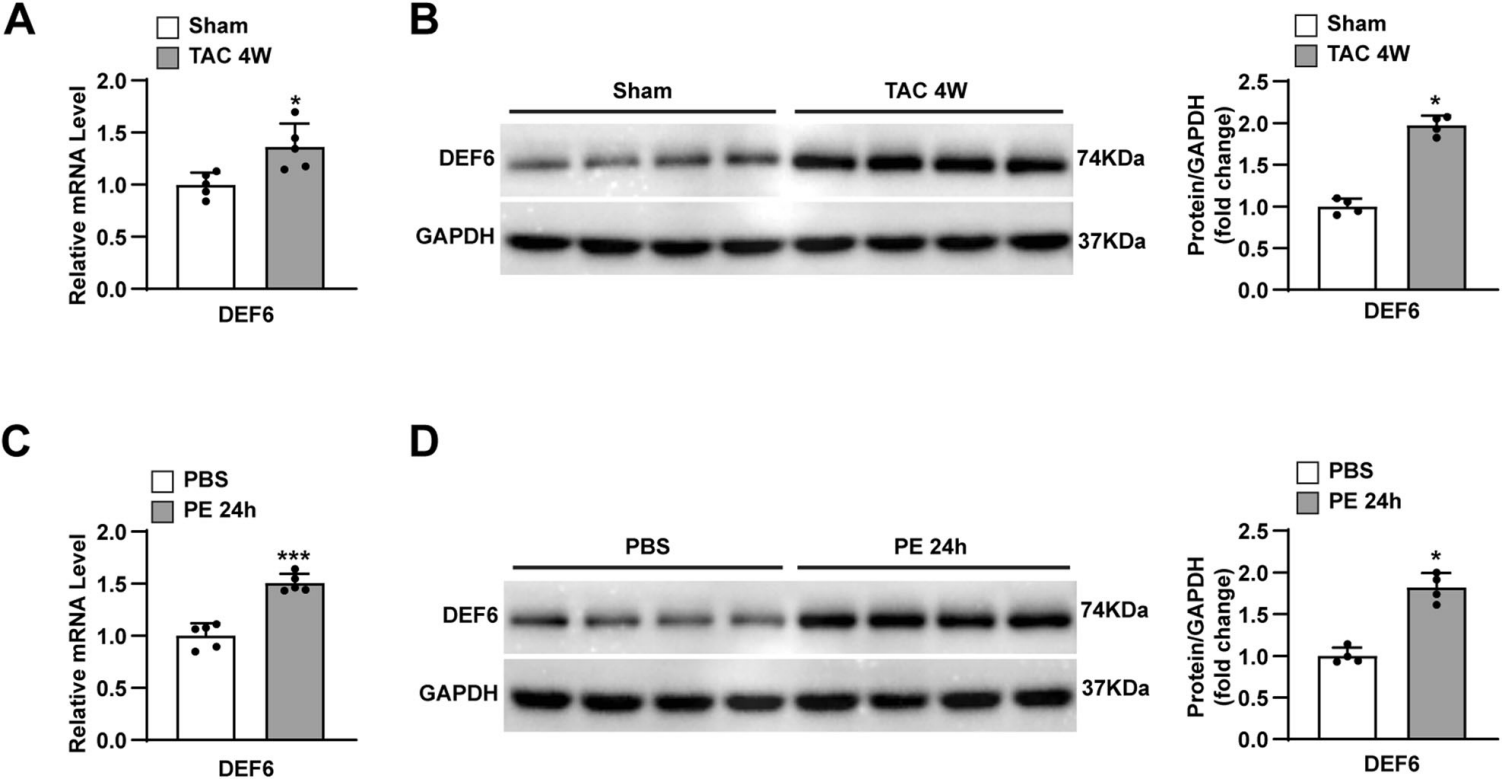
The expression of DEF6 is increased in hypertrophic hearts and cardiomyocytes. **A** mRNA levels of DEF6 in the LV myocardium of mice subjected to sham or 4 weeks of TAC surgery (*n* = 5). **B** Immunoblot analyses (left) and results of quantification (right) of DEF6 protein expression in the LV myocardium of mice subjected to sham or 4 weeks of TAC surgery (*n* = 4). **C** mRNA levels of DEF6 in NRCMs administrated with PBS or 24 h of PE (50 μM) (*n* = 5). **D** Immunoblot analyses (left) and results of quantification (right) of DEF6 protein expression in NRCMs administrated with PBS or 24 h of PE (*n* = 4). ^*^P < 0.05, ^***^P < 0.001 vs. sham or PBS. Data are displayed as mean ± SD. Statistical analysis were conducted by two-tailed Student’s *t* test (**A**, **C**) or Mann–Whitney *U* test (**B**, **D**).

Abundant expression of DEF6, which plays a crucial role in adaptive and innate immunity, has been described in the immune system, especially in T cells [8, 16]. The highly enriched expression of DEF6 in the heart also implies that DEF6 may have important roles in heart disease [26]. To further elucidate the impact of DEF6 on pathological cardiac hypertrophy, we initially determined the expression profile of DEF6 in ventricular samples of C57BL/6J wild-type (WT) mice processed with 1 week, 2 weeks, 4 weeks of TAC or sham surgery. According to Fig. 1A, B, and Fig. S1A, the mRNA and protein expression levels of DEF6 were remarkably enhanced after pressure overload in TAC-treated group compared with the control, and the protein expression levels of DEF6 increased with time after TAC. In parallel, we cultured NRCMs and treated them with PE (50 μM) or PBS as a control for 24 h to determine the expression profile of DEF6 in hypertrophic cardiomyocytes. Similar to in vivo results, the in vitro findings uncovered that DEF6 expression levels were greater in PE-treated NRCMs than in PBS-treated controls (Fig. 1C, D). This expression alteration implied that DEF6 might be implicated in pathological cardiac hypertrophy.

### Ablation of DEF6 mitigates TAC-induced cardiac hypertrophy

**Fig. 2:**
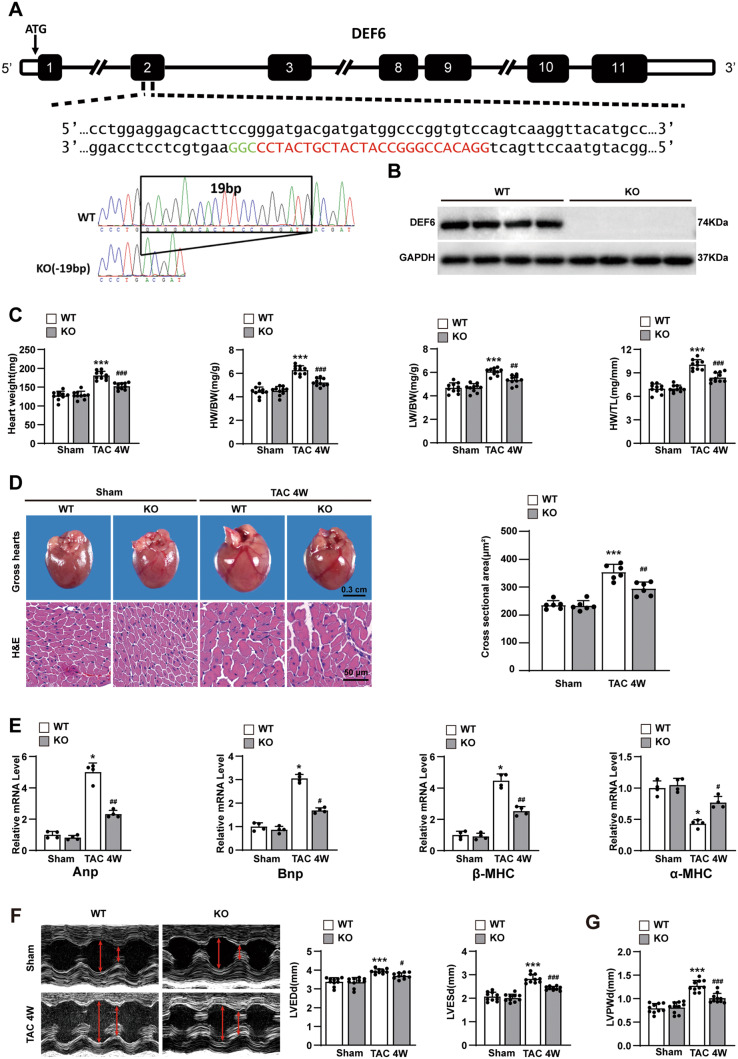

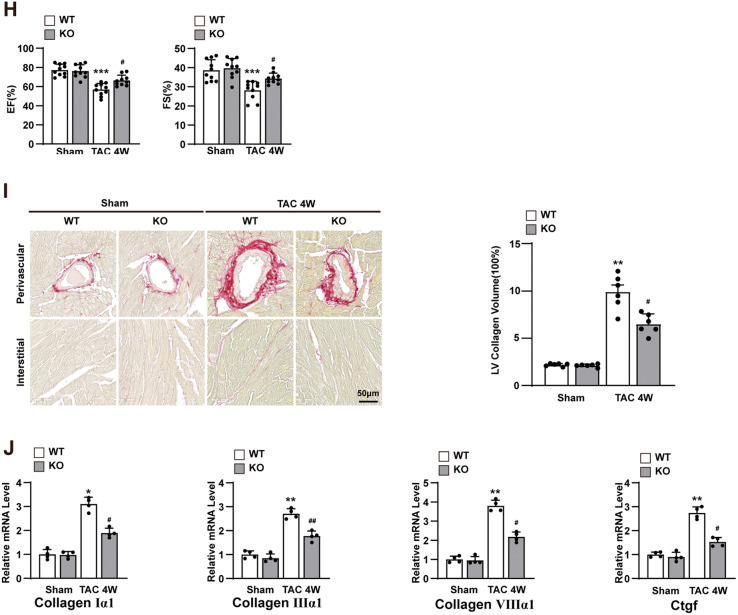
Ablation of DEF6 mitigates TAC-induced cardiac hypertrophy. **A** Strategy to construct KO mice and the sequencing results of WT and KO mice. **B** Protein levels of cardiac DEF6 in WT and KO mice (*n* = 5). **C** Comparisons of HW, HW/BW, LW/BW, and HW/TL in WT and KO mice subjected to sham or 4 weeks of TAC surgery (*n* = 10). **D** Left, gross hearts and H&E-stained LV sections of each groups. Scale bars, 0.3 cm and 50 μm, respectively. Right, Comparisons of cardiomyocyte cross-sectional area from groups (*n* = 6). **E** RT-PCR analyses of the hypertrophic markers in the indicated groups (*n* = 4). **F**–**H** Comparisons of the LVEDd, LVESd, LVPWd, FS, and EF values in WT and KO mice subjected to sham or 4 weeks of TAC surgery (*n* = 10). **I** Left, PSR-stained LV sections in WT and KO mice subjected to sham or 4 weeks of TAC surgery. Scale bars, 50 μm. Right, comparisons of LV collagen volume between groups (*n* = 6). **J** RT-PCR analysis of the fibrotic markers in each groups (*n* = 4). ^*^P < 0.05, ^**^P < 0.01, ^***^P < 0.001 vs. WT sham, ^#^*P* < 0.05, ^##^*P* < 0.01, ^###^*P* < 0.001 vs. WT TAC. Data are displayed as mean ± SD. Statistical analysis were conducted by One-way ANOVA (**C**, **D**, **F**–**I**) or Kruskal–Wallis test (**E**, **J**).

The above findings prompted the investigation into the impact of DEF6 on cardiac hypertrophy. To achieve this goal, we constructed a global DEF6-knockout (KO) mouse strain (Fig. 2A), and Western blot assay confirmed the deficiency of DEF6 in DEF6-KO mice (Fig. 2B).

We then subjected the KO and WT mice to TAC or sham. Four weeks later, the hypertrophic responses of the indicated groups were analyzed. At the basal level, DEF6 deficiency caused no apparent abnormalities; however, compared with the control condition, it exerted a remarkable ability to protect against pressure overload-induced increases in heart mass and cardiomyocyte size, as indicated by the decreased heart weight (HW), HW/body weight (BW), HW/tibia length (TL), gross heart sizes, and cardiomyocyte cross-sectional area values in KO mice (Fig. 2C, D). The lung weight (LW)/BW, a classic indicator of pulmonary congestion resulting from LV dysfunction, was also reduced in the KO mice (Fig. 2C). Accordingly, mRNA levels of Anp, Bnp, and β-MHC (hypertrophic markers) were all reduced in KO mice, while those of an antihypertrophic marker α-MHC were increased concurrently (Fig. 2E). In parallel with their alleviated cardiac hypertrophy, KO mice also displayed less ventricular expansion and better cardiac contractile function than their control counterparts, as manifested by decreases in LVEDd, LVESd, LVPWd, and increases in FS and EF (Fig. 2F–H).

We explored the impact of DEF6 ablation on pressure overload-induced cardiac fibrosis using PSR staining. Four weeks after TAC, KO mice exhibited lower LV collagen volumes than WT mice, indicating reduced cardiac fibrosis (Fig. 2I). These changes were in accordance with the decreased mRNA levels of the cardiac fibrosis markers collagen Iα1, collagen IIIα1, collagen VIIIα1, and connective tissue growth factor (Ctgf) (Fig. 2J).

### Overexpression of DEF6 aggravates TAC-induced cardiac hypertrophy

**Fig. 3:**
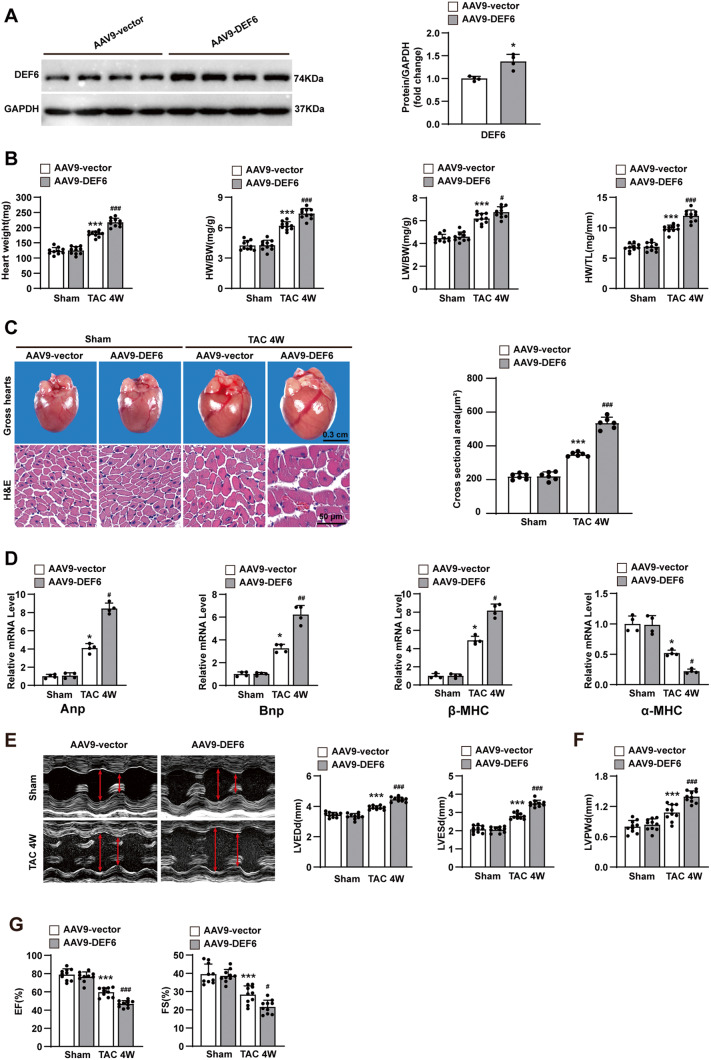

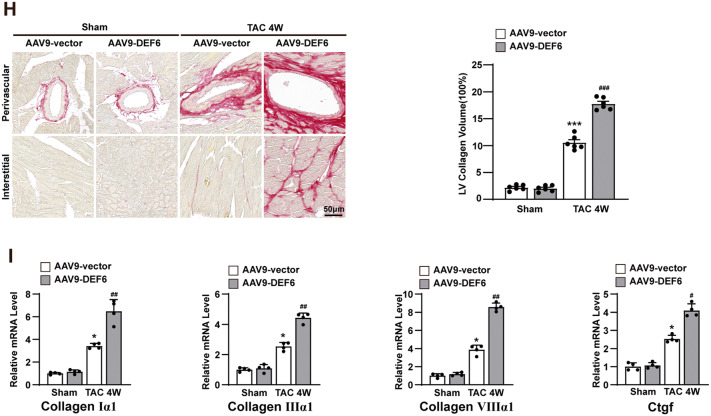
Overexpression of DEF6 aggravates TAC-induced cardiac hypertrophy. **A** mmunoblot analyses (left) and results of quantification (right) of DEF6 protein expression in the hearts of mice injected with AAV9-vector or AAV9-DEF6 (*n* = 4). **B** Comparisons of HW, HW/BW, LW/BW, and HW/TL in AAV9-vector- and AAV9-DEF6-infected mice subjected to sham or 4 weeks of TAC surgery (*n* = 10). **C** Left, gross hearts and H&E-stained LV sections of each groups. Scale bars, 0.3 cm and 50 μm, respectively. Right, Comparisons of cardiomyocyte cross-sectional area between groups (*n* = 6). **D** RT-PCR analyses of the hypertrophic markers in each groups (*n* = 4). **E**–**G** Comparisons of LVEDd, LVESd, LVPWd, FS, and EF in AAV9-vector- and AAV9-DEF6-infected mice subjected to sham or 4 weeks of TAC surgery (*n* = 10). **H** Left, PSR-stained LV sections in AAV9-vector- and AAV9-DEF6-infected mice subjected to sham or 4 weeks of TAC surgery. Scale bars, 50 μm. Right, comparisons of LV collagen volume between groups (*n* = 6). **I** RT-PCR analyses of the fibrotic markers in each groups (*n* = 4). ^*^P < 0.05, ^**^P < 0.01^, ***^P < 0.001 vs. AAV9-vector or AAV9-vector sham, ^#^*P* < 0.05, ^##^*P* < 0.01, ^###^*P* < 0.001 vs. AAV9-vector TAC^.^ Data are displayed as mean ± SD. Statistical analysis were conducted by Mann–Whitney *U* test (**A**) or One-way ANOVA (**B**, **C**, **E**–**H**) or Kruskal–Wallis test (**D**, **I**).

To explore the overall auxo-action of DEF6 in pathological cardiac hypertrophy, cardiomyocyte-specific DEF6-overexpressing mice (AAV9-DEF6) were constructed via tail vein injection of an AAV9 vector containing the DEF6 gene controlled by the cardiomyocyte-specific promoter cTNT. Mice injected with an empty AAV9 vector (AAV9-vector) were used as controls. The cardiomyocyte-specific overexpression of DEF6 was confirmed by Western blot (Fig. 3A, Fig. S1C).

DEF6-overexpressing (AAV9-DEF6) and control (AAV9-vector) mice underwent TAC or sham surgery and were analyzed 4 weeks later. Under basal conditions (sham), AAV9-DEF6 mice displayed no differences from AAV9-vector mice with regard to cardiac structure and contractile function. However, DEF6 overexpression significantly deteriorated pressure overload-induced cardiac hypertrophy and pulmonary congestion, as shown by increments in HW, HW/BW, LW/BW, HW/TL (Fig. 3B), gross heart sizes, and cardiomyocyte cross-sectional area versus the control (AAV9-vector) group (Fig. 3C). Furthermore, the increase in LV dimensions and reduction in cardiac contractility were remarkably exacerbated in AAV9-DEF6 mice, as indicated by echocardiographic analysis (LVEDd, LVESd, LVPWd, FS and EF; Fig. 3E–G). Accordingly, exacerbated cardiac fibrosis was detected in the LV myocardium of AAV9-DEF6 mice compared to the AAV9-vector mice after 4 weeks of TAC (Fig. 3H).

Coinciding with the progression of cardiac hypertrophy and fibrosis, DEF6 overexpression remarkably enhanced the mRNA levels of the cardiac hypertrophic and fibrotic markers (Fig. 3D, I). Conversely, the expression levels of the antihypertrophic marker α-MHC were lower in the LV myocardium of AAV9-DEF6 mice than in those of AAV9-vector mice after 4 weeks of TAC stimulation. All these data support that DEF6 contributes significantly to the development of pathological cardiac hypertrophy.

### DEF6 exacerbates PE-induced cardiomyocyte hypertrophy

**Fig. 4:**
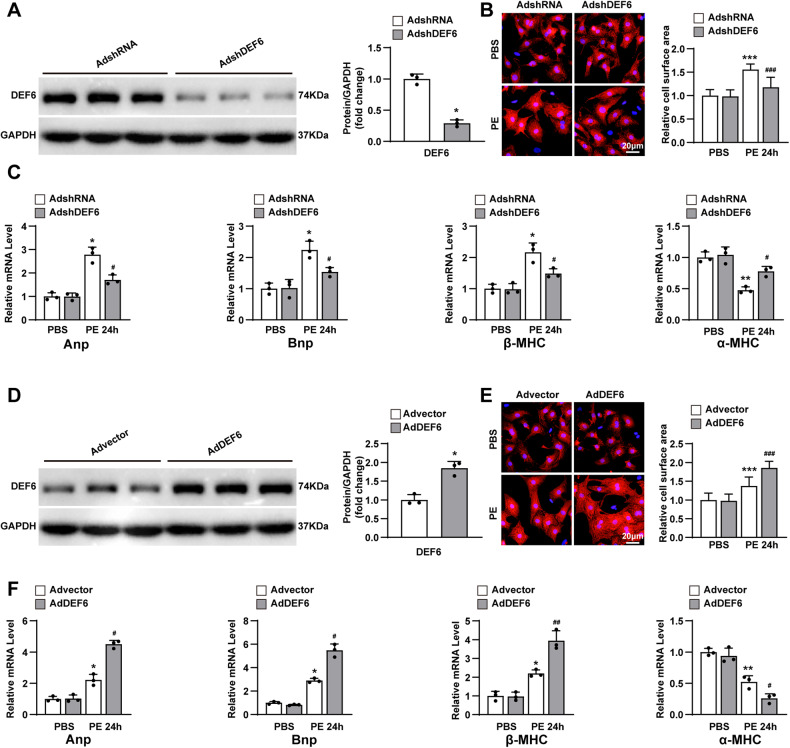
DEF6 exacerbates PE-induced cardiomyocyte hypertrophy. **A** Immunoblot analyses (left) and results of quantification (right) of DEF6 protein expression in cultured NRCMs infected with AdshRNA or AdshDEF6 (*n* = 3). **B** Left, immunofluorescence staining (α-actinin, red) in cultured NRCMs infected with AdshRNA or AdshDEF6 and administrated with PBS or 24 h of PE. Scale bar, 20 μm. Right, comparisons of the cardiomyocyte surface areas in cultured NRCVs of each groups (*n* ≥ 48 cells per group). **C** RT-PCR analysis of the hypertrophic markers in cultured NRCVs of each groups (*n* = 3). **D** Immunoblot analyses (left) and results of quantification (right) of DEF6 protein expression in cultured NRCVs infected with Advector or AdDEF6 (*n* = 3). **E** Left, immunofluorescence staining (α-actinin, red) in cultured NRCMs infected with Advector or AdDEF6 and administrated with PBS or 24 h of PE. Scale bar, 20 μm. Right, comparisons of the cardiomyocyte surface areas in cultured NRCVs of each groups (*n* ≥ 48 cells per group). **F** RT-PCR analyses of the hypertrophic markers in cultured NRCVs of each groups (*n* = 3). ^*^P < 0.05, ^**^P < 0.01^, ***^P < 0.001 vs. AdshRNA or AdshRNA PBS or Advector or Advector PBS, ^#^*P* < 0.05, ^##^*P* < 0.01, ^###^*P* < 0.001 vs. AdshRNA PE or Advector PE. Data are displayed as mean ± SD. Statistical analysis were conducted by two-tailed Mann*–*Whitney *U* test (**A**, **D**) or One-way ANOVA (**B**, **E**) or Kruskal–Wallis test (**C**, **F**).

The above in vivo data prompted further exploration about whether the hypertrophic effect of DEF6 acts mainly on cardiomyocytes. Therefore, further assays were conducted on cultured NRCMs that were infected with adenoviral vector containing short hairpin RNA against DEF6 (AdshDEF6) to knock down DEF6 or adenoviral vector containing DEF6 (AdDEF6) to overexpress this gene. In addition, an adenovirus encoding empty short hairpin RNA (AdshRNA) and an adenovirus encoding the empty vector (Advector) were used as controls. The adenovirus-mediated expression modification of DEF6 was verified by Western blot (Fig. 4A, D). After treated with either PE (50 μM) or PBS for 24 h, the cardiomyocytes immunostained with anti-α-actinin were photographed to measure cardiomyocyte surface areas. No differences with statistical significance in cell surface area were found among the groups under basal conditions (PBS treatment). Nevertheless, DEF6 knockdown ameliorated the cardiomyocyte hypertrophy, reduced hypertrophic markers expression, and enhanced antihypertrophic marker expression induced by PE administration compared to the control condition (AdshRNA) (Fig. 4B, C). Conversely, DEF6 overexpression worsened PE-induced cardiomyocyte hypertrophy (Fig. 4E, F). The data from this in vitro study support the finding that DEF6 can exacerbate PE-induced cardiomyocyte hypertrophy by directly regulating cardiomyocytes.

### DEF6 enhances hypertrophic stress-induced activation of MEK-ERK cascade

**Fig. 5:**
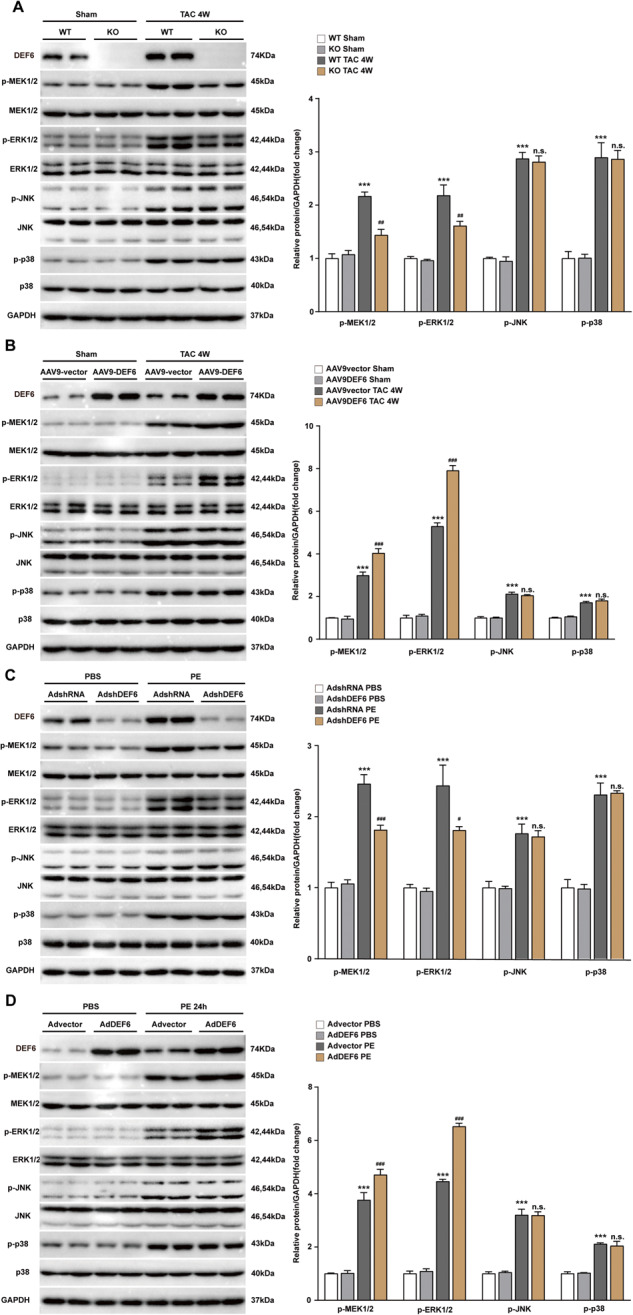
DEF6 enhances hypertrophic stress–induced activation of MEK-ERK signaling pathways. **A** Immunoblot analyses (left) and results of quantification (right) of the phosphorylated MEK1/2 (p-MEK1/2), ERK1/2 (p-ERK1/2), p38 (p-p38), and JNK (p-JNK) in heart tissue from WT and KO mice subjected to 4 weeks of sham or TAC surgery (*n* = 3). **B** Immunoblot analyses (left) and results of quantification (right) of the activated MAPK cascade (same as A) in heart tissue in AAV9-vector and AAV9-DEF6 mice subjected to 4 weeks of sham or TAC surgery (*n* = 3). **C** Immunoblot analyses (left) and results of quantification (right) of the activated MAPK cascade (same as A) in cultured NRCMs infected with AdshRNA or AdshDEF6 and administrated with 24 h of PBS or PE (*n* = 3). **D** Immunoblot analyses (left) and results of quantification (right) of the activated MAPK cascade (same as A) in cultured NRCMs infected with Advector or AdDEF6 and administrated with 24 h of PBS or PE (*n* = 3). ^***^P < 0.001 vs. WT Sham or AAV9-vector sham or AdshRNA PBS or Advector PBS, ^#^*P* < 0.05, ^##^*P* < 0.01, ^###^*P* < 0.001 vs. WT TAC or AAV9-vector TAC or AdshRNA PE or AdVector PE, and n.s. indicates no significance vs. WT TAC or AAV9-vector TAC or AdshRNA PE or AdVector PE. Data are displayed as mean ± SD. Statistical analysis were conducted by Kruskal–Wallis test.

Mitogen-activated protein kinase (MAPK) signaling is crucial for cardiac hypertrophy provoked by pressure overload or other pathological stimulation [1, 27–29]. To uncover the mechanisms by which DEF6 exerts its hypertrophic impact, we initially detected the status of MAPK cascade, including the protein levels of total and activated MEK1/2 (MAPK1/2), extracellular signal-regulated kinase 1/2 (ERK1/2), P38, and c-Jun N-terminal kinase (JNK). The Western blot results displayed that the protein levels of phosphorylated MEK1/2, ERK1/2, p38, and JNK were markedly greater in TAC-treated mice or PE-treated cardiomyocytes than in their corresponding sham- or PBS-treated controls (Fig. 5A–D). The phosphorylation of ERK1/2 and MEK1/2 provoked by TAC surgery was abated by KO of DEF6 but dramatically intensified by overexpression of DEF6 in the heart, while p38 and JNK were not obviously influenced by DEF6 regulation (Fig. 5A, B). Similarly, the in vivo data revealed that DEF6 knockdown ameliorated the activation of MEK1/2 and ERK1/2 in cultured NRCMs challenged with PE without changing p38 and JNK activation (Fig. 5C). In contrast, overexpression of DEF6 enhanced the activation of MEK1/2 and ERK1/2 but not that of p38 and JNK (Fig. 5D).

### Prohypertrophic effect of DEF6 depends on Rac1-MEK-ERK signaling

As an upstream activator of MEK-ERK signaling, RAC1 has also been implicated in pathological cardiac hypertrophy and activated by DEF6 in the immune system and cell morphology modification [7, 30, 31]. Therefore, we hypothesized that DEF6 may regulate pathological cardiac hypertrophy through MEK-ERK signaling in an RCA1-dependent manner.

**Fig. 6:**
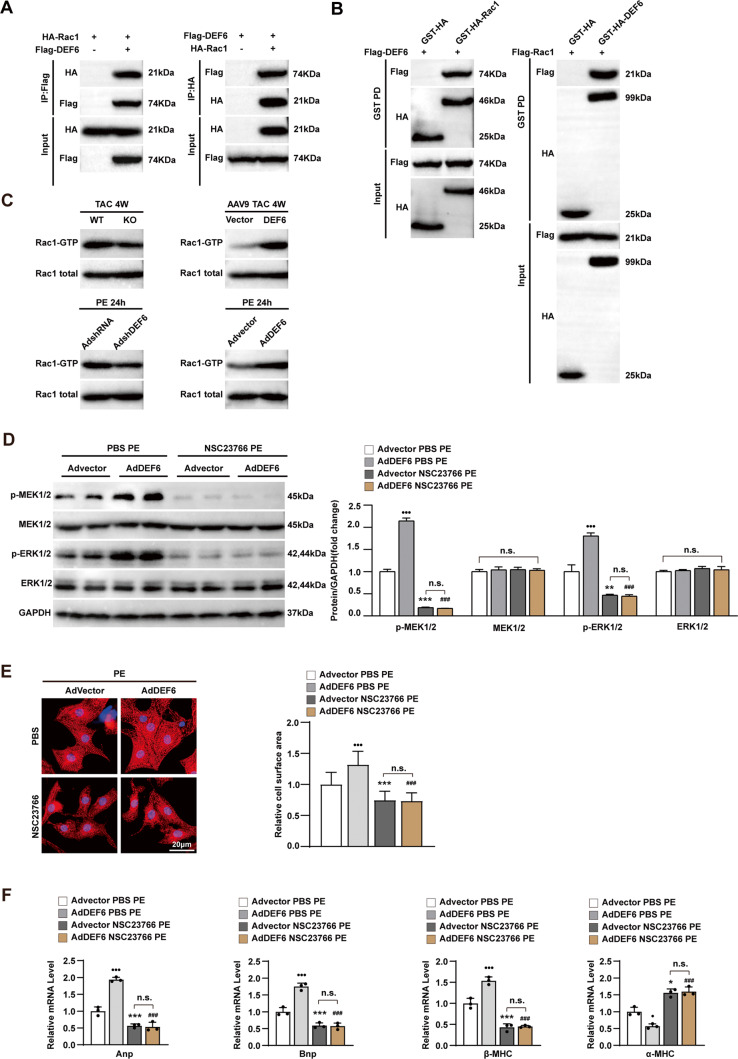

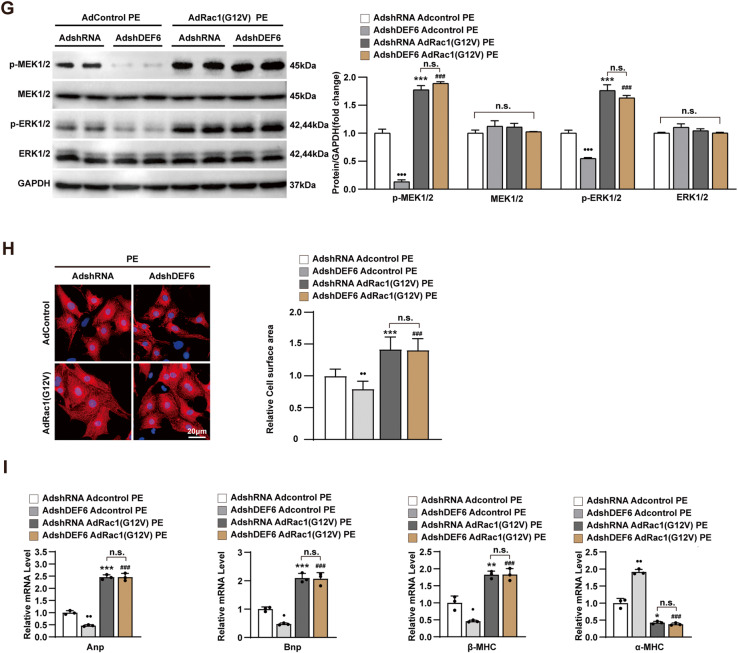
Prohypertrophic effect of DEF6 depends on Rac1-MEK-ERK signaling. **A** Co-IP of DEF6 was performed with anti-Flag and probed by Western blots with anti-HA (left); Co-IP of Rac1 was performed with anti-HA and probed by Western blots with anti-Flag (right). **B** In vitro GST pulldown assays for the interaction of purified Flag-DEF6 and GST-HA-Rac1 (left), as well as Flag-Rac1 and GST-HA-DEF6 (right). **C** The activity of Rac1 changes in the same direction as the DEF6 expression. **D** Immunoblot analyses of total and activated MEK1/2, ERK1/2 in cultured NRCMs infected with Advector or AdDEF6 and treated with PBS or NSC23766 (50 μM, 24 h) under 24 h of PE treatment (50 μM) (*n* = 3). **E** Immunofluorescence staining (α-actinin, red) (left) and comparison of cardiomyocyte surface areas (right) of NRCMs infected with Advector and AdDEF6 and treated with PBS or NSC23766 (50 μM, 24 h) under 24 h of PE treatment (50 μM). (n ≥ 48 cells per group). **F** RT-PCR analysis of the hypertrophic markers in cultured NRCVs of each groups (*n* = 3). **G** Immunoblot analyses of total and activated MEK1/2, ERK1/2 in cultured NRCMs infected with AdshRNA or AdshDEF6 and with Adcontrol or AdRac1(G12V) under PE 24 h of PE treatment (50 μM) (*n* = 3). **H** Immunofluorescence staining (α-actinin, red) (left) and comparison of cardiomyocyte surface areas (right) of NRCMs infected with the indicated adenovirus and administrated with 24 h of PE (50 μM) (n ≥ 48 cells per group). **I** RT-PCR analysis of the hypertrophic markers in cultured NRCVs of each groups (*n* = 3). ^•^*P* < 0.05, *P* < 0.01,^•^*P* < 0.001 vs. Advector PBS PE or AdshRNA Adcontrol PE, ^*^P < 0.05, ^**^P < 0.01, ^***^P < 0.001 vs. Advector PBS PE or AdshRNA Adcontrol PE, ^###^*P* < 0.001 vs. AdDEF6 PBS PE or AdshDEF6 Adcontrol PE, and n.s. indicates no significance. Data are displayed as mean ± SD. Statistical analysis were conducted by Kruskal–Wallis test.

We initially explored whether DEF6 and RAC1 directly act upon each other. Flag-tagged DEF6 and HA-tagged RCA1 were co-translated in HEK293T cells for Co-IP experiment. As shown in Fig. 6A, immunoblotting with an antibody against HA (anti-HA) after IP with an antibody against Flag (anti-Flag) indicated that DEF6 coimmunoprecipitated with RCA1. Conversely, IP with anti-HA and immunoblotting with anti-Flag suggested that RAC1 immunoprecipitated with DEF6. To rule out the possibility that both proteins bind indirectly, we then performed an in vitro GST pulldown assay. A GST fusion protein and a corresponding target protein were expressed and purified in bacteria. After incubation of two cell extracts, the DEF6-RCA1 binding complex was validated by immunoblotting. The results revealed that DEF6 and RAC1 extracted from cell lysate could bind to and precipitate each other but not GST alone (Fig. 6B). Collectively, these findings verify that DEF6 can directly interact with RAC1.

Then, we assessed the influence of DEF6 expression alteration on the activities of Rac1 after hypertrophic stimulations. Both in vivo and in vitro data exhibited that DEF6 knockdown reduced the expression of activated Rac1(Rac1 bound to GTP, Rac1-GTP) and DEF6 overexpression remarkably enhanced activities of Rac1 in response to TAC or PE treatment (Fig. 6C). Those findings supported that DEF6 can enhance activities of Rac1 through increasing binding activity between Rac1 and GTP in cardiac hypertrophy.

Many previous studies have confirmed the involvement of Rac1-MEK-ERK1/2 signaling cascade in cardiac hypertrophy [31, 32]. Therefore, the next step was to find out whether RAC1 could affect activity of MEK-ERK signaling in PE-triggered cardiomyocyte hypertrophy, and whether this kind of change could affect the regulatory effect of DEF6 in this context. For this purpose, the Rac1 inhibitor NSC23766 (PBS as control), an adenovirus encoding a constitutively active mutant of Rac1 (AdRac1(G12V)) and a control adenovirus (Adcontrol) were utilized for subsequent experiments. After 24 h treatment with NSC23766 or PBS, NRCMs were infected with AdDEF6 or Advector for 6 h, and then treated with PE for 24 h. As predicted, we observed that Rac1 inhibitor NSC23766 significantly rescued PE-triggered activation of MEK1/2 and ERK1/2 and even that promoted by DEF6 overexpression (Fig. 6D). The hypertrophic phenotype of NRCMs was evaluated via assessment of cardiomyocyte size and the mRNA levels of hypertrophic markers. As delineated in Fig. 6E, F, the data revealed that NSC23766 neutralized the auxo-action of DEF6 overexpression on the enlargement of cardiomyocyte size, the increment of the mRNA levels of Anp, Bnp, β-MHC, and the reductions in the mRNA levels of α-MHC induced by PE administration.

On the other hand, AdRac1(G12V) or Adcontrol was co-transfected with AdshDEF6 or AdshRNA into NRCMs before PE treatment (24 h). As expected, the Western blotting data also suggested that activation of MEK1/2 and ERK1/2 triggered by PE was inhibited by DEF6 knockdown but this ameliorative effect reversed by constitutively Rac1 activation (Fig. 6G). Meanwhile, we found that constitutively active Rac1 neutralized the ameliorating effects of DEF6 knockdown on the enlargement of cardiomyocyte size, the increment of the mRNA levels of Anp, Bnp, β-MHC, and the reductions in the mRNA levels of α-MHC induced by PE administration (Fig. 6H, I). To conclude, activation of Rac1-MEK-ERK signaling cascades is some of the mechanisms by which DEF6 accelerates the development of pathological cardiac hypertrophy.

## Discussion

The high incidence of heart failure necessitates illumination of the underlying mechanisms of pathological cardiac hypertrophy to identify useful therapeutic targets. In this context, the current study started from an observation of elevated expression levels of DEF6 in the hypertrophic myocardium and in hypertrophic cardiomyocytes. Our further experiments uncovered that DEF6 aggravated pathological hypertrophy, contractile dysfunction, fibrosis, and cardiomyocyte hypertrophy induced by pro-hypertrophic stimuli, but did not work under physiological conditions. A subsequent mechanistic study revealed that DEF6 execute its prohypertrophic effect by directly interacting with and activating RAC1, thereby activating downstream MEK-ERK1/2 signaling. Thus, we first demonstrated that the expression level of DEF6 is sensitive to the severity of pathological cardiac hypertrophy and DEF6 can remarkably exacerbates the pressure overload-induced cardiac hypertrophy.

The significantly elevated expression levels of DEF6 were observed in hypertrophic heart and cardiomyocytes, however, the mechanism of this expression alteration induced by hypertrophic stimuli remains unknown. Plenty of evidence has demonstrated that a number of GEFs can be activated by hypertrophic stimuli (PE, Ang II, endothelin-1, Stretch and others) through GPCRs, receptor tyrosine kinases, and other membrane receptors to activate Rac1 and eventually lead to cardiac hypertrophy [32–35]. Since DEF6 is also a GEF of Rac1, whether the activation of membrane receptors by pro-hypertrophic stimuli contributes to the increase in DEF6 expression provoked by pressure overload and PE remains to be further elucidated.

Mechanistically, our research reveals that MAPK signaling participates in the effects of DEF6 on cardiac hypertrophy. MAPK signaling pathway, which includes three members, ERKs, JNKs, and p38, plays an extraordinarily vital role in cardiac hypertrophy provoked by overload and other pathological stimuli [1, 27–29]. ERK1/2 is a serine/threonine kinase that mainly responds to stimulation by growth factors and can activate other serine/threonine kinases and several transcription factors to contribute to cell differentiation, proliferation, and survival [36, 37]. It has been verified that ERK1/2 can be activated by receptor agonists as well as cell stretching in cultured cardiomyocytes and by acute pressure overload in the mouse myocardium [38]. Constitutively active MEK1, the upstream activator of ERK1/2, induces cardiomyocyte hypertrophy in vitro, and that is attenuated in the presence of dominant-negative MEK1 [37, 39]. Our results supported the above researches and uncovered that DEF6 expedites the progression of pathological cardiac hypertrophy and cardiomyocyte hypertrophy also by activating MEK1/2-ERK1/2 cascade.

Ras-related C3 botulinum toxin substrate GTPases (also called Racs) belongs to the Rho small GTPase family. There are three Rac isoforms: Rac1 is widely expressed; Rac2 is expressed specifically in hematopoietic cells; and Rac3 has been reported to be expressed in the brain, lungs, liver, and pancreas [40]. Numerous studies have verified the crucial role of Rac1 in the pathogenesis of cardiac hypertrophy. Many In vitro studies have shown that overexpression of a constitutively active isoform of Rac1(G12V) in NRCMs leads to morphological and biochemical hypertrophic effects similar to those in the PE-induced cardiomyocytic response, whereas overexpressing a dominant-negative mutant Rac1(N17), inhibits PE-induced cardiomyocytic hypertrophy and myocardial oxidative stress [30, 32]. In vivo, overexpression of Rac1(G12V) in the mouse heart results in a cardiomyopathy phenotype characterized by hypertrophy or dilation [41]. In contrast, gene transfer of the dominant-negative mutant Rac1(N17) suppresses pressure overload-stimulated cardiac hypertrophy [34], and cardiomyocyte-specific deficiency of Rac1 in mice reduces angiotensin-II-induced oxidative stress and myocardial hypertrophy [40]. Many previous studies had connected Rac1-mediated cardiac hypertrophy and the MEK1/2-ERK1/2 signaling pathway activation. Clerk et al. found that the hypertrophic agonists PE and endothelin 1 (ET-1) can activate Rac1 and then result in cardiac hypertrophy through regulation of ERK and probably JNK, but not p38; in addition, the upregulation of ERK activity and hypertrophy induced by these agonists are inhibited by Rac1(N17) [32]. A recent study revealed that STEAP3 ameliorates pathological cardiac hypertrophy by blocking the Rac1-MEK-ERK1/2 signaling cascade [31]. In our mechanistic exploration, we found that DEF6 could directly interacted with Rac1 and enhance activities of Rac1 through increasing the binding ability of Rac1 with GTP in pathological cardiac hypertrophy. These results implied that DEF6 activates Rac1 in a GEF’s manner. The further rescue experiments using Rac1 inhibitor NSC23766 treatment and AdRac1(G12V) infection verified the requirement of Rac1 and MEK1/2-ERK1/2 activation for DEF6-mediated pathological cardiac hypertrophy. This result is in line with previous researches about the role of Rac1-dependent MEK-ERK1/2 signaling cascade in cardiac hypertrophy [31, 32]. Of note, rescue experiments confirmed that inhibiting or continuously activating Rac1 could not only completely reverse the effects of DEF6 overexpression or silence on the phenotype of cardiomyocyte hypertrophy and the activity of MEK-ERK1/2 signaling, but also created addition effects compared to basic condition, suggesting that Rac1 activation is necessary and sufficient for the development of pathological cardiac hypertrophy. A large body of evidences revealed the involvements of Rac1 in the activation of JNK and P38 signaling pathways [42–44]. However, there was no changes about phosphorylation of p38 and JNK in our study. A probable explanation is that the MAPK signaling pathways are regulated in a cell type- and stimulus type-specific manner.

**Fig. 7:**
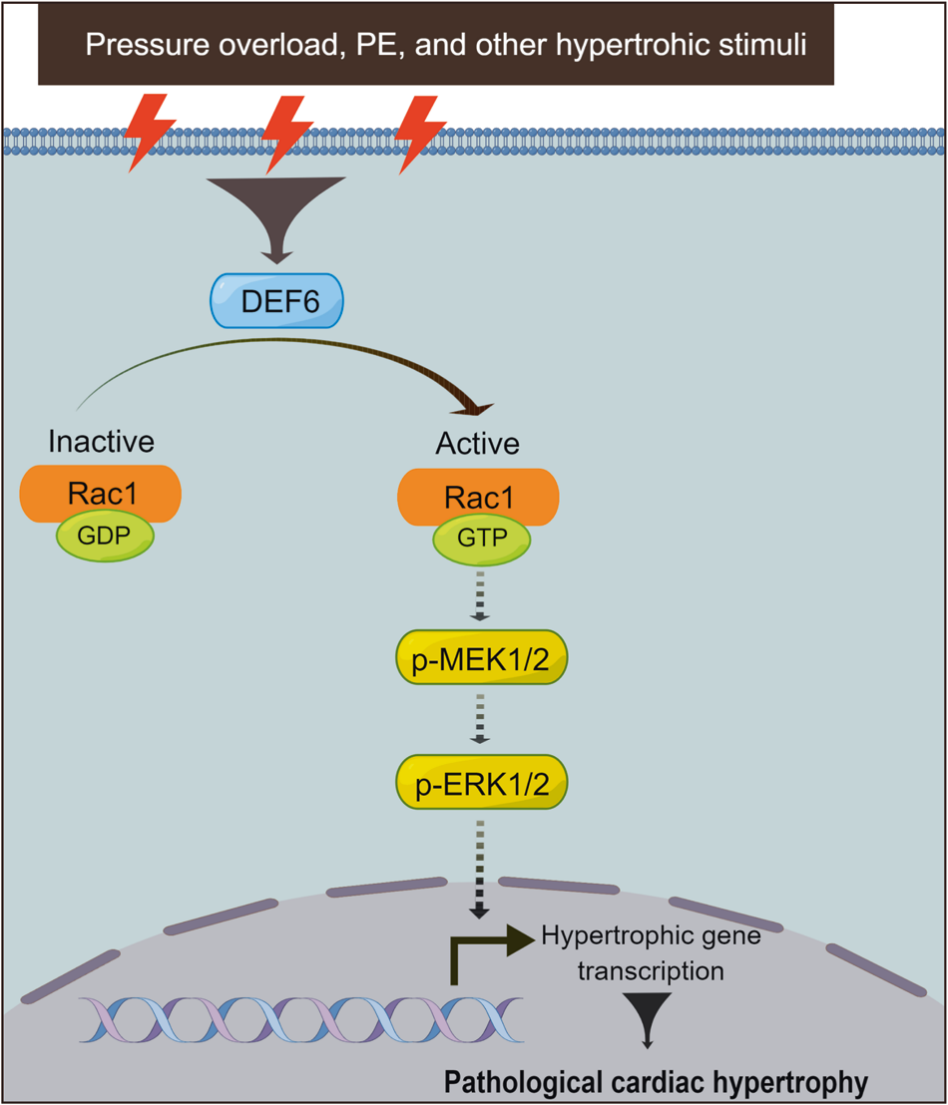
The role and signaling of DEF6 in cardiac hypertrophy. DEF6 activate Rac1 in a GEF-manner, promoting cardiac hypertrophy via MEK1/2-ERK1/2 pathway.

Previous study on DEF6 mainly focused on its function in immunity and cancers. However, our current research first reveals the deleterious regulatory effects of DEF6 in cardiac hypertrophy through the Rac1-MEK1/2-ERK1/2 pathways (Fig. 7). It reveals that Rac1 is a key intermediate for the pro-hypertrophic effect of DEF6, but the exact mechanisms how DEF6 activate Rac1 is still unknown. In addition, global DEF6 knockout mice were used in our study instead of the cardiomyocyte-specific type. To address this limitation, a rescue experiment is necessary to be performed where DEF6 levels are recovered with AAV9 in the future. Thus, further studies are necessary to elucidate these issues. In summary, the current study provides convincing evidence that DEF6 exacerbates the development of cardiac remodeling by activating Rac1-MEK-ERK1/2 signaling. These findings may aid in the exploration of novel treatment strategies for heart failure involving targeting of DEF6.

## Materials and Methods

### Animals

Animals were applied to experiments with the approval of the Ethics Committee of Shengjing Hospital of China Medical University (NO. 2022PS458K). Animal experiments in this study were performed according to the Guide for the Care and Use of Laboratory Animals.

Production of DEF6-knockout (KO) mice: An online CRISPR design tool (http://chopchop.cbu.uib.no/) was utilized to design guiding sequences targeting the second exon of the mouse DEF6 gene (guideRNA1 target site: GGACACCGGGCCATCATCGTCATCC-CGG), and pUC57-sgRNA (Addgene, 51132) was used to generate the DEF6-sgRNA expression vector. The in vitro transcription products of the DEF6-sgRNA expression vector and Cas9 expression vector pST1374-CAS9 (Addgene, 44758) were purified and mixed. In the next step, fertilized eggs collected from C57BL/6 mice were microinjected with this mixture using a FemtoJet 5247 microinjection system and subsequently transplanted into pseudopregnant foster mothers' oviducts. After 19-21 days, F0-generation mice were born. Two weeks later, genomic samples were obtained from the toes of F0 mice, and the genotypes were tested using the following pair of primers: F : 5′-CCGGATGCAGAAAAGCAACC-3′, R : 5′-CGCGGGAGCTAAGAGAGATG-3′.

### AAV9 virus-based gene modification in mice

AAV9-DEF6 and AAV9 control vectors (AAV9-vector) were constructed as described previously [45]. Briefly, the mouse DEF6 encoding sequence was cloned into a pAAV vector with a cardiomyocyte-specific cTNT promoter. AAV-293 cells were cotransfected with the recombinant helper plasmid and pAAV-RC, and the recombinant vectors were purified by iodixanol gradient centrifugation after 3 days. Finally, the titers of the AAV9-DEF6 and AAV9-vector were measured using real-time quantitative PCR (qPCR). Fourteen days before TAC surgery, randomly selected male mice with C57BL/6J backgrounds were injected in the tail vein with 7.5×10^11^ viral genomes (vg) of the AAV9-DEF6 or AAV9-vector. The relevant primers: DEF6 F: 5′-CACGCTTAACTAGCTAGCCACCATGGCCCTGCGCAAGGAG-3′,DEF6 R: 5′-AGCTCCGCTTCCACGCGTCTTATCGTCGTCATCCTTGTAATCATTCCCTGGTGCTGGATCCAG-3′.

### Mouse TAC surgery

In this study, we performed TAC surgery to generate pressure overload-provoked model of cardiac hypertrophy as described previously [28]. Nine- to eleven-week-old male mice weighing 25.5–27 g were selected for the experiment and randomly divided into groups. Briefly, after anesthetization, TAC surgery was performed by ligating the aortic arch over a 26-gauge blunt needle using 7-0 silk sutures, producing a reduction in luminal diameter. Sham control mice were treated with the same procedure but without aortic constriction. The pressure overload induced by TAC was validated by echocardiographic measurement of the pressure gradient resulting from aortic constriction.

### Echocardiographic analyses

For echocardiographic analysis, animals were anesthetized using isoflurane (1.5–2%). Cardiac function evaluation and hemodynamic analysis were performed by transthoracic echocardiography with an Animal Ultrasound Imaging System (FUJIFILM VISUALSONICS, VEVO2100) equipped with a 30 MHz probe (MS400). The left ventricular (LV) end-diastolic diameter (LVEDd), LV end-systolic diameter (LVESd), and LV posterior wall diastolic thickness (LVPWd) were measured for three beats from each projection and averaged. Cardiac function was determined by calculating the LV fractional shortening (FS) and LV ejection fraction (EF). Echocardiography was conducted by investigators blinded to the study

### Histological analyses

The mouse hearts were excised and stopped in the diastolic period with a 10% KCl solution after 4 weeks of sham or AB surgery and then processed by 10% formalin and embedded in paraffin. For morphological analysis and collagen deposition detection, the paraffin blocks were cut into five- micrometer-thick slices, which were then stained with hematoxylin (Servicebio, G1004) & eosin (BASO, BA-4024) (H&E) or picrosirius red (PSR; Hedebiotechnology, 26357-02). Image-Pro Plus 6.0 (IPP 6.0) was used for image analysis.

### Culture of primary neonatal rat cardiomyocytes (NRCMs) and adenovirus transfection

The rat full-length DEF6 cDNA or the constitutively active mouse Rac1(G12V) mutant was cloned into a replication-deficient adenoviral vector that contained a cytomegalovirus promoter ((AdDEF6) or AdRac1(G12V)). The control was an empty adenoviral vector (Advector). Adenoviral constructs contains short hairpin RNA against rat DEF6 (AdshDEF6) was used to silence DEF6 gene expression, and AdshRNA was utilized as a nontargeting control. Then, we isolated NRCMs from the hearts of neonatal sprague-dawley (SD) rats, NRCMs were seeded in culture plates coated with gelatin at 1.8×10^6^ cells/well as previously described [28]. The cardiomyocytes were cultured in DMEM/F12 medium (Gibco, C11330) added with 10% fetal bovine serum (FBS), 5-bromodeoxyuridine (0.1 mM), and 1% penicillin/streptomycin for 24 h. The NRCMs were infected with adenoviruses at a multiplicity of infection (MOI) of 100 for 6 h. Subsequently, the medium was replaced with serum-free DMEM/F12, and 12 h later, the cardiomyocytes were stimulated with phosphate-buffered saline (PBS) or PE (50 μM) for 24 h. For rescue experiments, the cardiomyocytes were treated with Rac1 inhibitor NSC23766 (50 μM, 24 h)(S8031, Selleck) before adenovirus infection [31]. The relevant primers are as follows:

AdDEF6-Rat-F: GGCTAGCGATATCGGATCCGCCACCATGGCCCTGCGCAAGGAG

AdDEF6-Rat -R: CGTCCTTGTAATCACTAGTATTTTCTGGCGCTGCATCCAG

AdshDEF6-Rat-F: CCGGGGTCCTTCACATCCCTCATGACTCGAGTCATGAGGGATGTGAAGGACCTTTTTG

AdshDEF6-Rat-R: AATTCAAAAAGGTCCTTCACATCCCTCATGACTCGAGTCATGAGGGATGTGAAGGACC

AdRac1(G12V)-Mouse-F: GGCTAGCGATATCGGATCCGCCACCATGCAGGCCATCAAGTGTGTGGTGGTGGGAGACGTAGCTGTTGG

AdRac1(G12V)-Mouse-R: CGTCCTTGTAATCACTAGTCAACAGCAGGCATTTTCTCTTCCTC

### Immunofluorescence staining

After stimulation with PE, the NRCM surface area was measured by immunofluorescence. The NRCMs were processed in 4% formaldehyde for 30 min and then washed more than three times. After that, the cardiomyocytes were processed with 0.2% Triton X-100 in PBS for 5 min, incubated with α-actinin antibody (anti-α-actinin, Merck Millipore, 05-384, 1:100 dilution) and then labeled with a fluorescent secondary antibody (Invitrogen, A21202, 1:200 dilution). The nucleus of the cells was stained with DAPI. Image analysis of NRCM surface area was conducted with IPP 6.0.

### Coimmunoprecipitation (Co-IP)

Briefly, cultured HEK293T cells were co-transfected with plasmids expressing the fusion proteins and then lysed. The supernatant obtained from the lysates was incubated with Protein G-conjugated agarose beads (Bestchrom, AA104307) and corresponding antibodies against the label (anti-HA or anti-Flag) overnight at temperature of 4 °C. After washing by IP buffer, the proteins were eluted and then collected from the agarose beads. Finally, the proteins were tested and analyzed by Western blotting. The primers of the involved fusion proteins are presented in Supplementary Table 1.

### GST pulldown

Briefly, HEK293T cells were transfected with plasmids expressing the fusion proteins GST-HA-Rac1, GST-HA-DEF6, Flag-Rac1, and Flag-DEF6 and then lysed. After purification, the fusion proteins GST-HA-Rac1 and Flag-DEF6 or Flag-Rac1 and GST-HA-DEF6 were mixed and then incubated overnight at 4 °C. The immunocomplex was washed and collected with the corresponding buffers (20 mM Tris-HCl; 150 mM NaCl; 0.2% Triton X-100; and 2X SDS). Finally, the proteins were tested and analyzed by Western blotting.

### Rac1 activity assay

Rac1 activation “pull-down” assay (Upstate Biotechnology) was used to assess the activities of Rac1 [46]. According to the manufacturer’s instructions, cardiac tissue or NRCMs were processed using the magnesium lysis buffer, and then co-incubated with glutathione beads which coupled with GST fusion protein containing the p21-binding domain of PAK1. Rac1 content was determined by immunoblotting.

### Western blot assay

Total proteins extraction were described as a previous study [28]. Detection of total protein extracts concentration was conducted utilizing a BCA Protein Assay Kit (Pierce, 23225). After being separated by SDS–PAGE, total protein extracts (50 µg) were transferred to 0.45 µm polyvinylidene fluoride membranes and detected with corresponding antibodies. The membranes were stained with peroxidase-conjugated secondary antibodies (Jackson ImmunoResearch) and then visualized with enhanced chemiluminescence (ECL) reagent (Bio-Rad, 1705062,) and a Bio-Rad ChemiDoc^TM^ XRS+ system (Bio-Rad). Glyceraldehyde-3-phosphate dehydrogenase (GAPDH) was used for normalization. Information on the antibodies is listed in Supplementary Table 2.

### Real-time polymerase chain reaction (RT–PCR)

TRIzol reagent (Invitrogen, 15596-026) was applied for total RNA extraction from cardiac tissue or NRCMs. Two micrograms of RNA were reverse-transcribed into first-strand cDNA using a Transcriptor First Strand cDNA Synthesis Kit (04896866001, Roche), and PCR amplification was performed using SYBR Green PCR Master Mix (04887352001, Roche). The target gene expression levels were normalized against GAPDH. The primers of the involved genes are presented in Supplementary Table 3.

### Statistical analysis

In our study, all data are described as the mean ± standard deviation (SD), and visualized using GraphPad Prism 8.0. Data analysis were performed using SPSS (version 26.0). In animal experiments, *n* ≥ 6 were used for pathology studies or functional analysis. In the cell experiments, at least 3 independent replications were performed. All data were subjected to the normality test. The means of 2-group samples were compared using two-tailed Student’s *t* tests (*n* ≥ 5) or Mann–Whitney *U* test (*n* ≤ 4) . One-way ANOVA with the Bonferroni test (for data with homogeneous variances, normal distribution, and *n* ≥ 5) or the Tamhane T2 test (for data with heterogeneous variances, normal distribution, and *n* ≥ 5) and Kruskal–Wallis test (for data with skewed distribution, or *n* ≤ 4) were used to test differences among more than 2 groups. *P* < 0.05 was considered to indicate statistical significance.

## Supplementary information

SUPPLEMENTARY FIGURE AND TABLE LEGENDS

Figure Supplementary1

Figure Supplementary2

Supplementary table 1, 2, 3

Original Data File

Original Data File

Reproducibility checklist

## Data Availability

The authors confirm that the data supporting the conclusions in the paper are presented in the article and its Supplementary Material. Additional data related to this paper are available from the corresponding author.
