# Supplementary material for: DEF6(differentially exprehomolog) exacerbates pathological cardiac hypertrophy via RAC1
Source: Cell Death Dis. 2023 Jul 31;14(7):483. doi: 10.1038/s41419-023-05948-0 (PMC10390462; doi:10.1038/s41419-023-05948-0)
Supplement: Supplementary file 1 — SUPPLEMENTARY FIGURE AND TABLE LEGENDS [file 41419_2023_5948_MOESM1_ESM.docx]

**SUPPLEMENTARY FIGURE LEGENDS**

**Fig. S1 A.** The protein level of DEF6 in mice heart tissues induced by Sham or TAC surgery for 1 week, 2 weeks, and 4 weeks. **B**. The heart rate of the mice between groups when EF was measured (n = 10). **C**. The protein level of DEF6 in heart, liver, kidney, and skeletal muscle tissues of mice infected with AAV9-vector and AAV9-DEF6. **D**. The heart rate of the mice between groups when EF was measured (n = 10). n.s. indicates no significance between groups.

**Fig S2,** Immunoblot analyses of the total MEK1/2, ERK1/2, p38, and JNK in heart tissue from groups (n = 3). n.s. indicates no significance between groups.

**SUPPLEMENTARY TABLE LEGENDS**

**Supplementary table 1**: The primers of the involved fusion proteins in Coimmunoprecipitation assay.

**Supplementary table 2**: Information on the antibodies used in this study.

**Supplementary table 3**: The primers of the involved genes in RT-PCR.
