# Supplementary figures and images for: DEF6(differentially exprehomolog) exacerbates pathological cardiac hypertrophy via RAC1
Source: Cell Death Dis. 2023 Jul 31;14(7):483. doi: 10.1038/s41419-023-05948-0 (PMC10390462; doi:10.1038/s41419-023-05948-0)

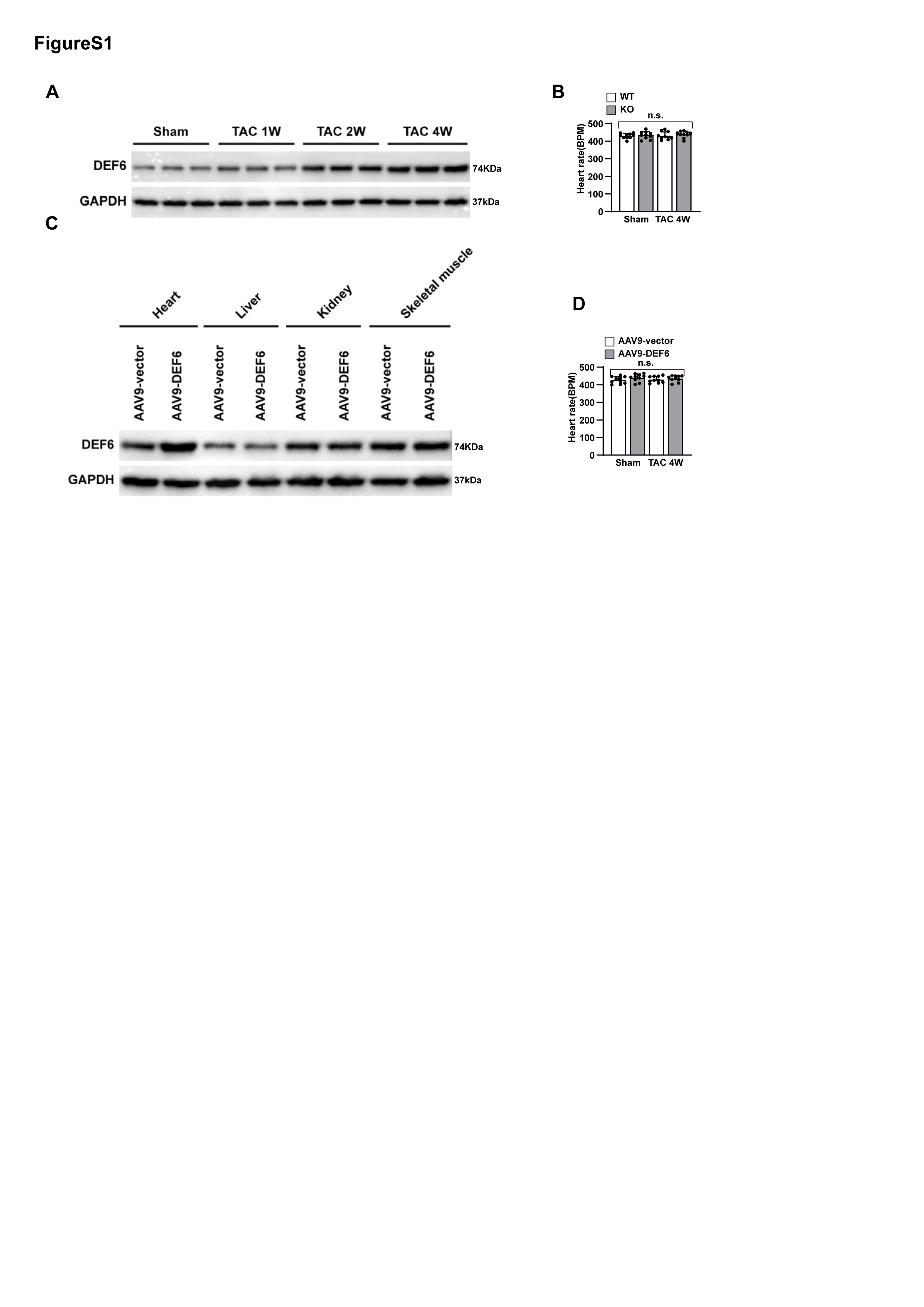

Supplement: Supplementary file 2 — Figure Supplementary1 [file 41419_2023_5948_MOESM2_ESM.tif]

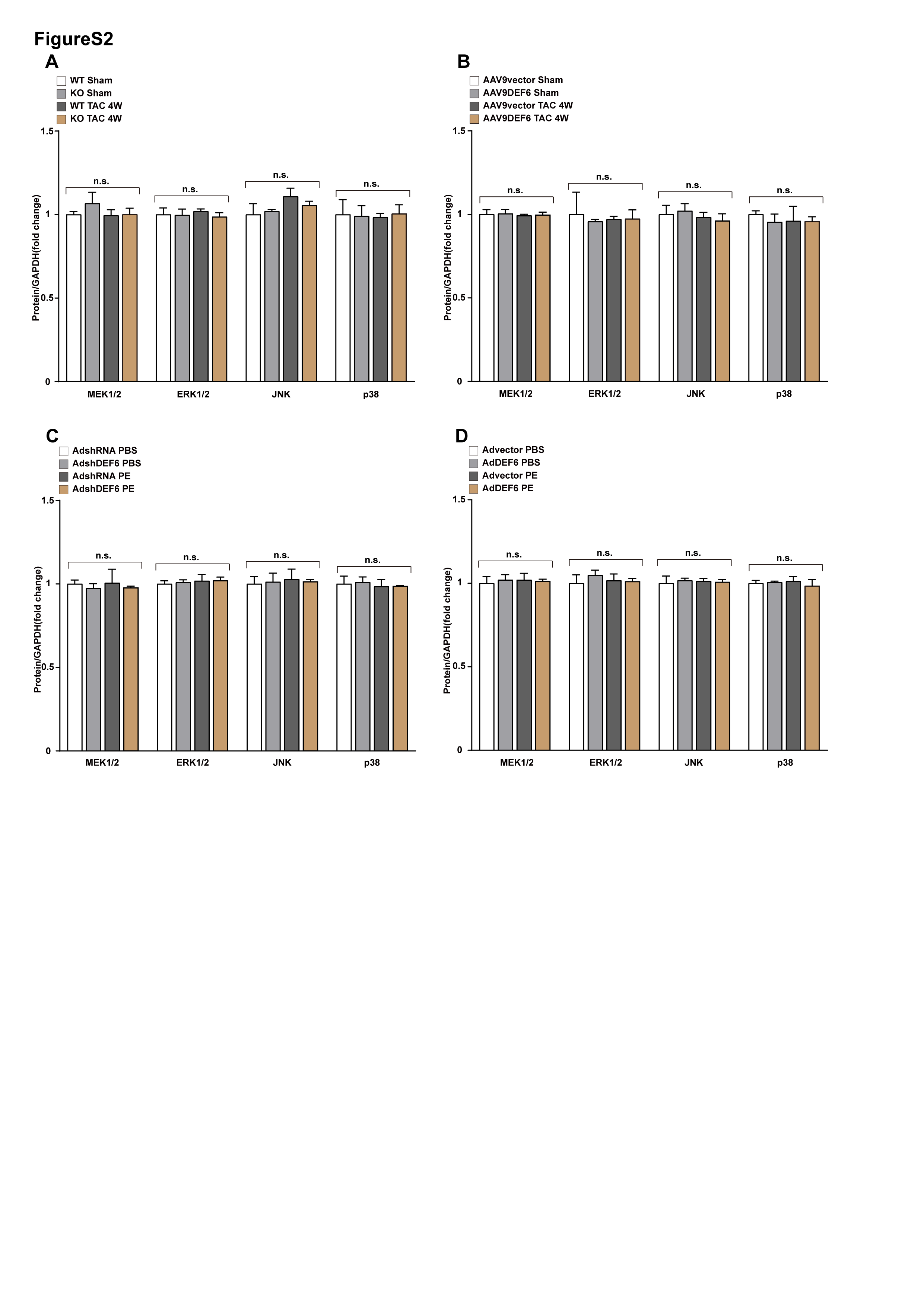

Supplement: Supplementary file 3 — Figure Supplementary2 [file 41419_2023_5948_MOESM3_ESM.tif]

FigureS1

A

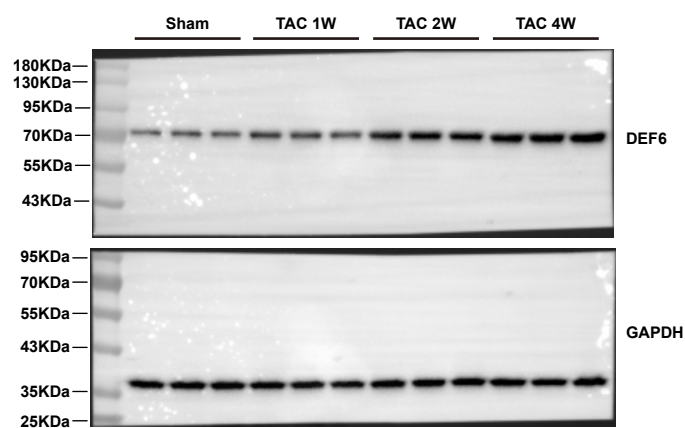

Supplement: Supplementary file 6 — Original Data File [file 41419_2023_5948_MOESM6_ESM.pdf]
