# Supplementary material for: DEF6(differentially exprehomolog) exacerbates pathological cardiac hypertrophy via RAC1
Source: Cell Death Dis. 2023 Jul 31;14(7):483. doi: 10.1038/s41419-023-05948-0 (PMC10390462; doi:10.1038/s41419-023-05948-0)
Supplement: Supplementary file 4 — Supplementary table 1, 2, 3 [file 41419_2023_5948_MOESM4_ESM.docx]

**Table**

**Supplementary table 1**

| Gene | Primer sequence(5’-3’) | |
| --- | --- | --- |
| HA-Rac1 | F | TCGGGTTTAAACGGATCCATGCAGGCCATCAAGTGTGTG |
|  | R | GGGCCCTCTAGACTCGAGTTACAACAGCAGGCATTTTCTCTTC |
| Flag-DEF6 | F | TCGGGTTTAAACGGATCCATGGCCCTGCGCAAGGAAC |
|  | R | GGGCCCTCTAGACTCGAGCTAATTTTCTGGTGCTGGATCCAG |
| HA-DEF6 | F | TCGGGTTTAAACGGATCCATGGCCCTGCGCAAGGAAC |
|  | R | GGGCCCTCTAGACTCGAGCTAATTTTCTGGTGCTGGATCCAG |
| Flag-Rac1 | F | TCGGGTTTAAACGGATCCATGCAGGCCATCAAGTGTGTG |
|  | R | GGGCCCTCTAGACTCGAGTTACAACAGCAGGCATTTTCTCTTC |

**Supplementary table 2**

| Anbibody | Manufacturer | Catalogue number | Source of species | Dilution |
| --- | --- | --- | --- | --- |
| DEF6 | ORIGENE | TA505321 | rabbit | 1:1000 |
| p-MEK1/2 | CST | 9154 | rabbit | 1:1000 |
| MEK1/2 | CST | 9122 | rabbit | 1:1000 |
| p-ERK1/2 | CST | 4370 | rabbit | 1:1000 |
| ERK1/2 | CST | 4695 | rabbit | 1:1000 |
| P-JNK | CST | 4668 | rabbit | 1:1000 |
| JNK | CST | 9252 | rabbit | 1:1000 |
| p-p38 | CST | 4511 | rabbit | 1:1000 |
| p38 | CST | 9212 | rabbit | 1:1000 |
| Flag | MBL | M185-3LL | rabbit | 1:1000 |
| HA | MBL | M180-3 | rabbit | 1:1000 |
| GAPDH | CST | 2118 | rabbit | 1:5000 |

**Supplementary table 3**

| Gene name | Forward primer (mouse) | | Reverse primer (mouse) |
| --- | --- | --- | --- |
| *DEF6* | CGAGCCAGGAGATAAGCGAC | | GGTGGTCGGAGCTAATTCCC |
| *Anp* | TCGGAGCCTACGAAGATCCA | | TTCGGTACCGGAAGCTGTTG |
| *Bnp* | GAAGGACCAAGGCCTCACAA | | TTCAGTGCGTTACAGCCCAA |
| *Myh7* | CAACCTGTCCAAGTTCCGCA | | TACTCCTCATTCAGGCCCTTG |
| *Myh6* | TCTGCCTACCTTATGGGGCT | | CGTACACTGACTTGGCCAGT |
| *Collagen Iα1* | TGCTAACGTGGTTCGTGACCGT | | ACATCTTGAGGTCGCGGCATGT |
| *Collagen IIIα1* | ACGTAAGCACTGGTGGACAG | | CCGGCTGGAAAGAAGTCTGA |
| *Ctgf* | TGACCCCTGCGACCCACA | | TACACCGACCCACCGAAGACACAG |
| *Collagen Ⅷα1* | | GCAACCAGGAGCAAAAGGTG | ATGCATACCTGGAGGACCCT |
| *Gapdh* | ACTCCACTCACGGCAAATTC | | TCTCCATGGTGGTGAAGACA |
|  |  | |  |
| Gene name | Forward primer (rat) | | Reverse primer (rat) |
| *DEF6* | GACGAGGATGTGGAGGCTG | | GCTGGAAGCCTGAGAAGGAG |
| *Anp* | AAAGCAAACTGAGGGCTCTGCTCG | | TTCGGTACCGGAAGCTGTTGCA |
| *Bnp* | TGCCCCAGATGATTCTGCTC | | TGTAGGGCCTTGGTCCTTTG |
| *Myh7* | AGTTCGGGCGAGTCAAAGATG | | CAGGTTGTCTTGTTCCGCCT |
| *Myh6* | CTCCAGGGGTGATGGACAAC | | CGATACCTCTGCCGGAAGTC |
| *Gapdh* | CAGTGCCAGCCTCGTCTCAT | | AGGGGCATCCACAGTCTTC |
