# Supplementary material for: DEF6(differentially exprehomolog) exacerbates pathological cardiac hypertrophy via RAC1
Source: Cell Death Dis. 2023 Jul 31;14(7):483. doi: 10.1038/s41419-023-05948-0 (PMC10390462; doi:10.1038/s41419-023-05948-0)

The original files of the western blot :

Fig1B

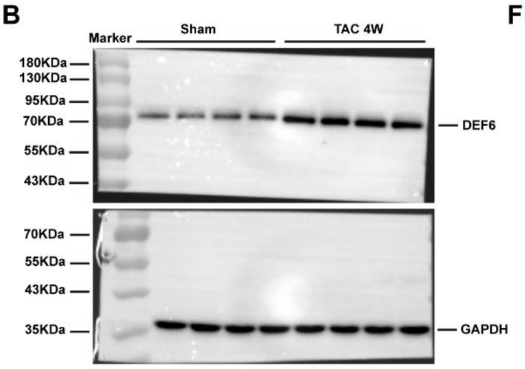

Fig1D

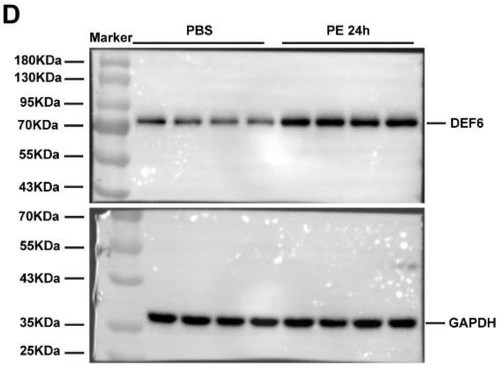

Fig2B

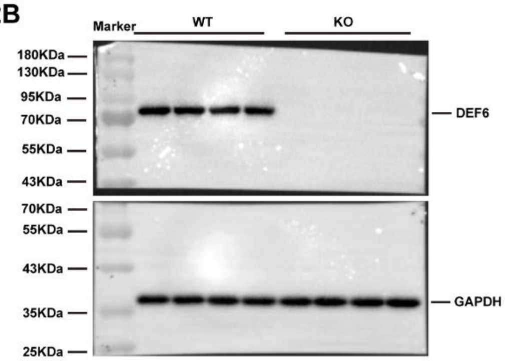

Fig3A

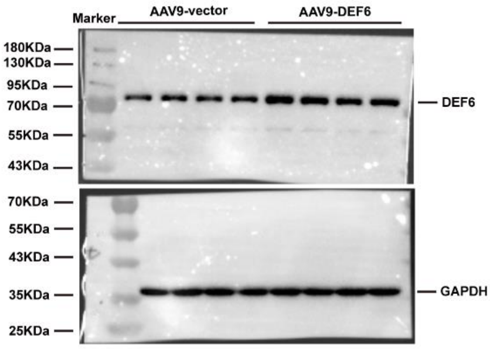

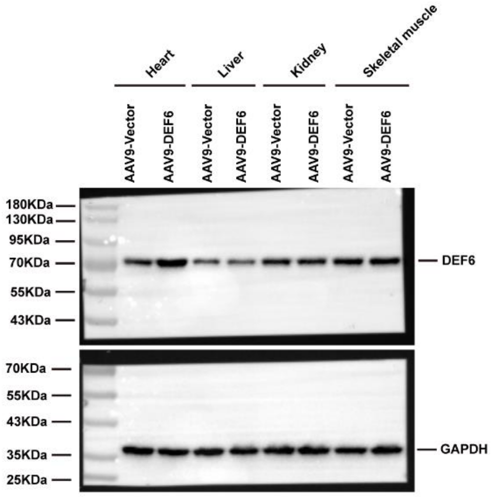

Fig4A

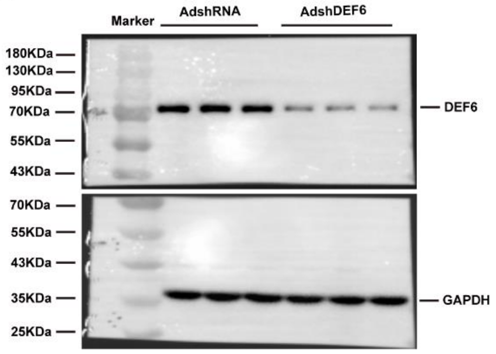

Fig4D

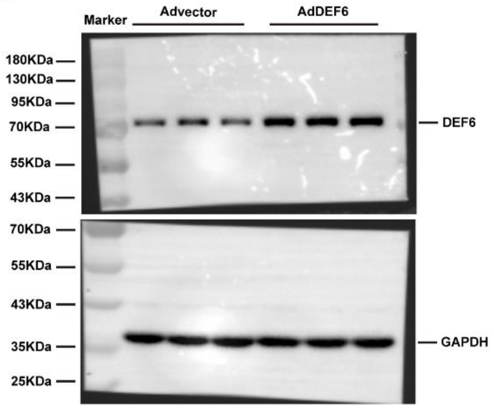

Fig5A

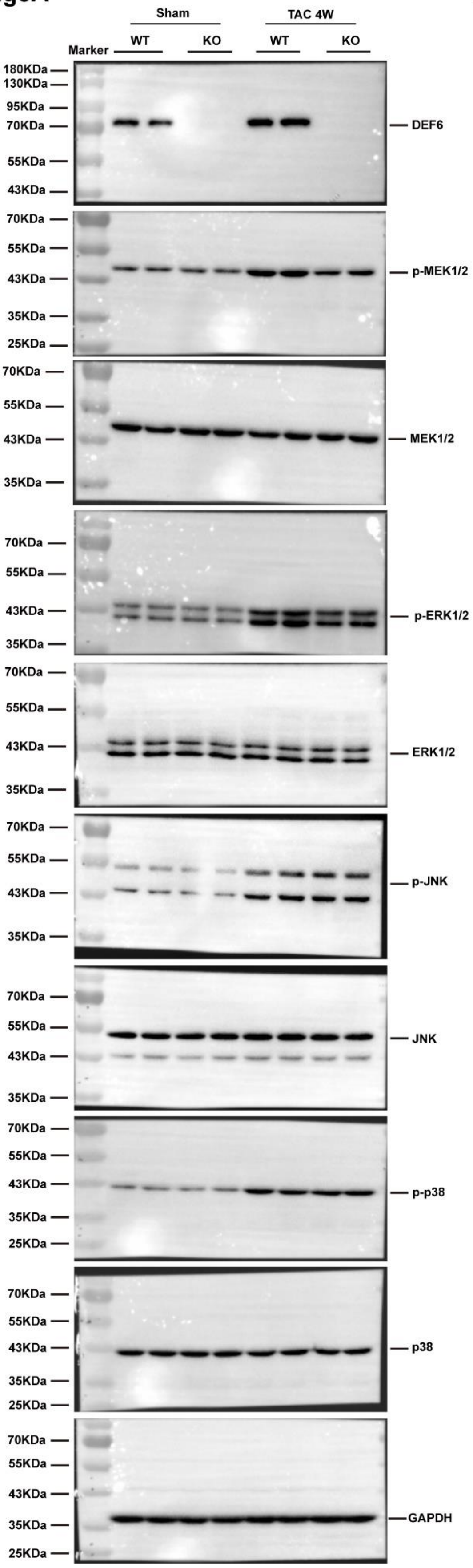

Sham
TAC 4W
Marker
WT
KO
WT
KO
180KDa
130KDa
95KDa
70KDa
55KDa
43KDa
35KDa
25KDa
DEF6
p-MEK1/2
MEK1/2
p-ERK1/2
ERK1/2
p-JNK
JNK
p-p38
p38
GAPDH

Fig5B

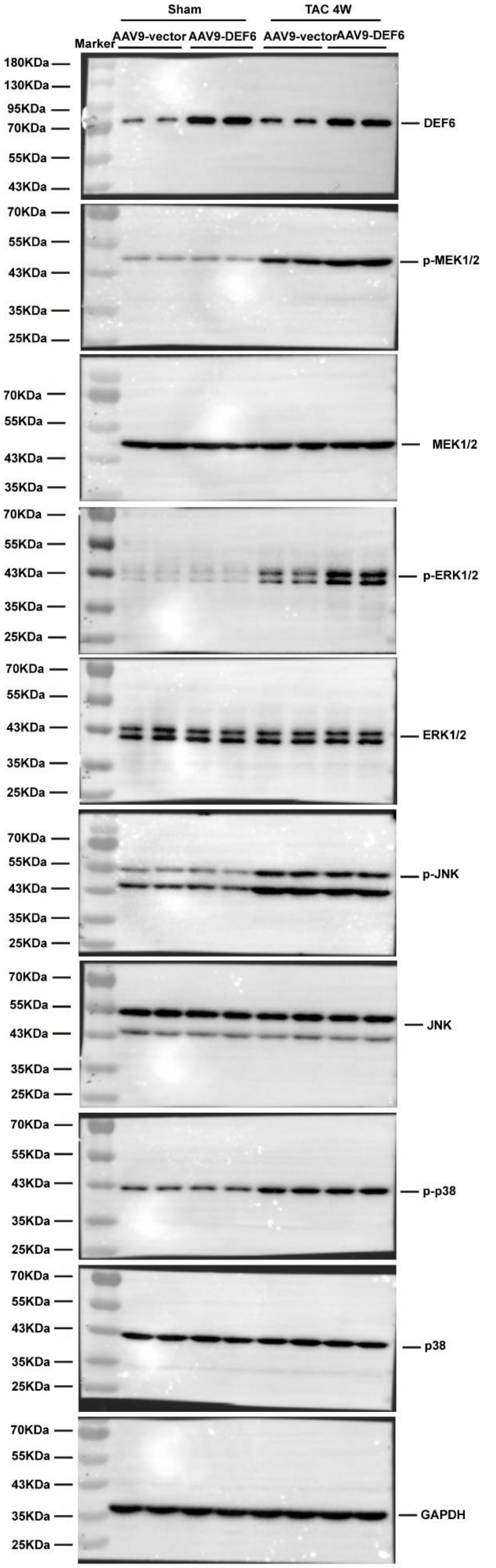

Sham
TAC 4W
Marker
AAV9-vector
AAV9-DEF6
AAV9-vector
AAV9-DEF6
180KDa
130KDa
95KDa
70KDa
55KDa
43KDa
35KDa
25KDa
DEF6
p-MEK1/2
MEK1/2
p-ERK1/2
ERK1/2
p-JNK
JNK
p-p38
p38
GAPDH

Fig5C

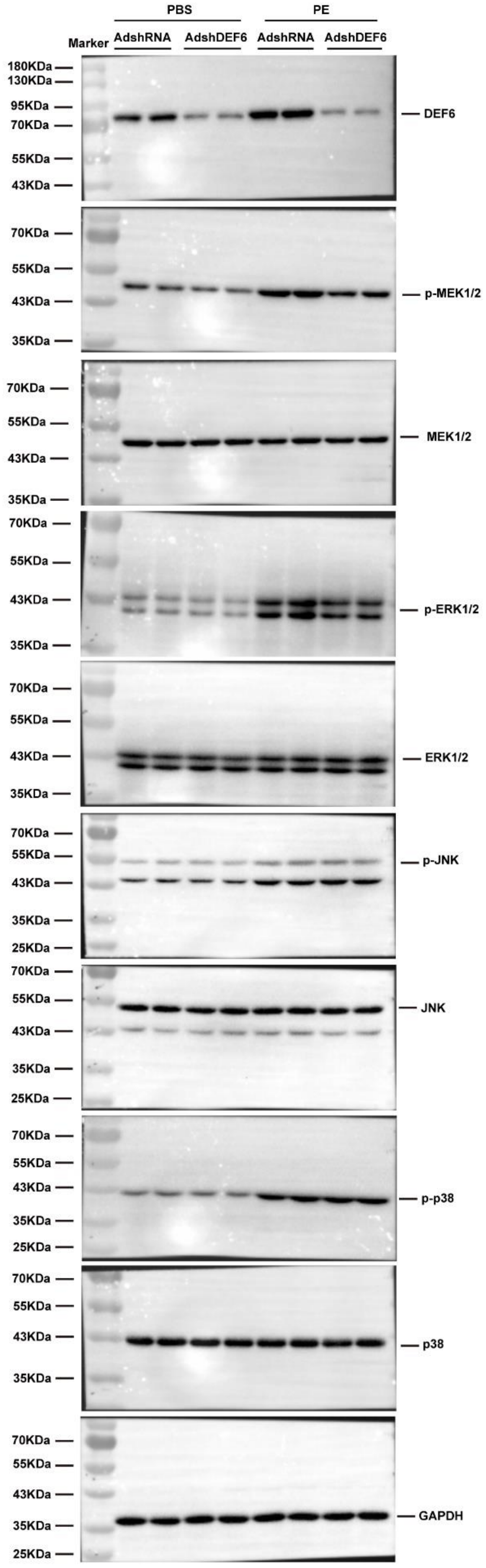

PBS
PE
Marker
AdshRNA
AdshDEF6
AdshRNA
AdshDEF6
180KDa
130KDa
95KDa
70KDa
55KDa
43KDa
35KDa
25KDa
DEF6
p-MEK1/2
MEK1/2
p-ERK1/2
ERK1/2
p-JNK
JNK
p-p38
p38
GAPDH

Fig5D

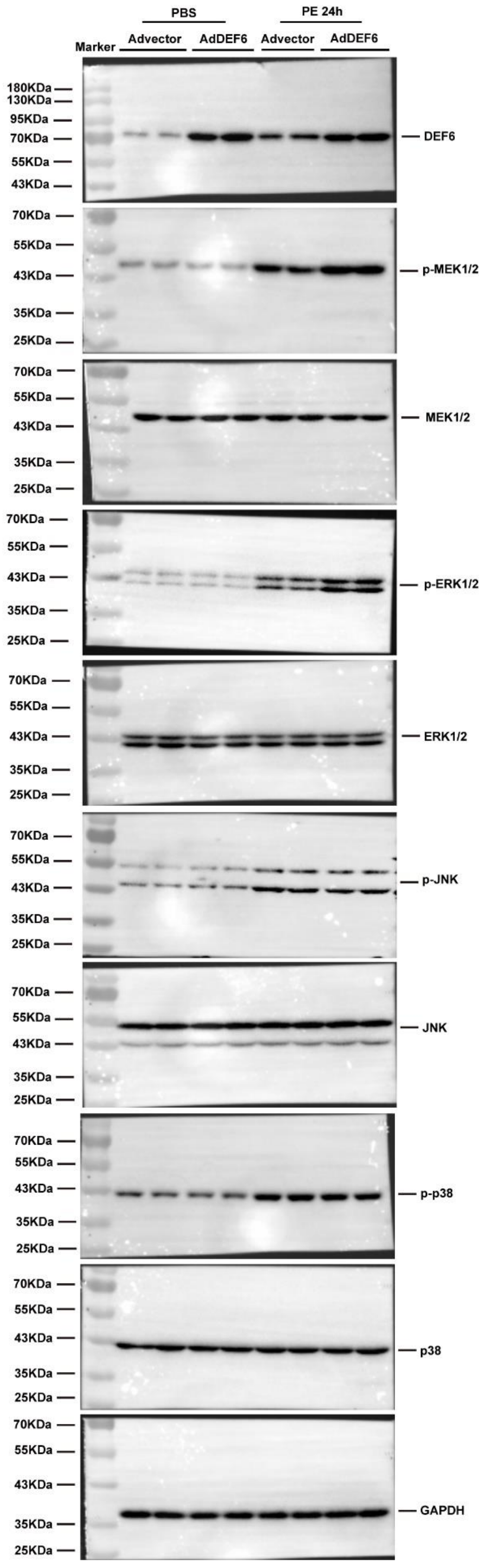

PBS
PE 24h
Marker
Advector
AdDEF6
Advector
AdDEF6
180KDa
130KDa
95KDa
70KDa
55KDa
43KDa
35KDa
25KDa
DEF6
p-MEK1/2
MEK1/2
p-ERK1/2
ERK1/2
p-JNK
JNK
p-p38
p38
GAPDH

Fig6A

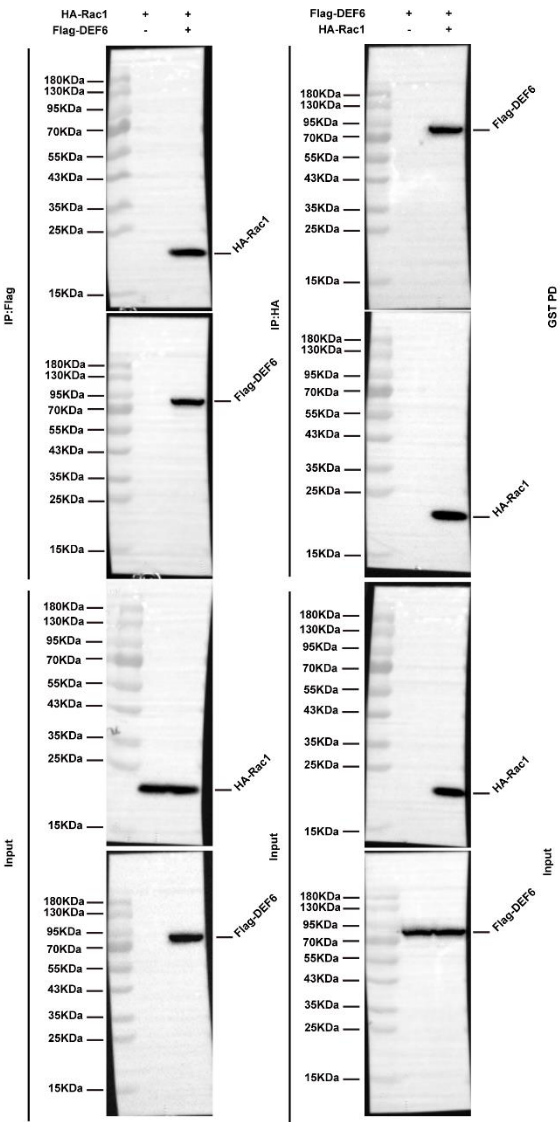
HA-Rac1 + +
Flag-DEF6 - +
IP:Flag
Input
HA-Rac1
Flag-DEF6
Flag-DEF6 + +
HA-Rac1 - +
IP:HA
180KDa
130KDa
95KDa
70KDa
55KDa
43KDa
35KDa
25KDa
15KDa

Fig6B

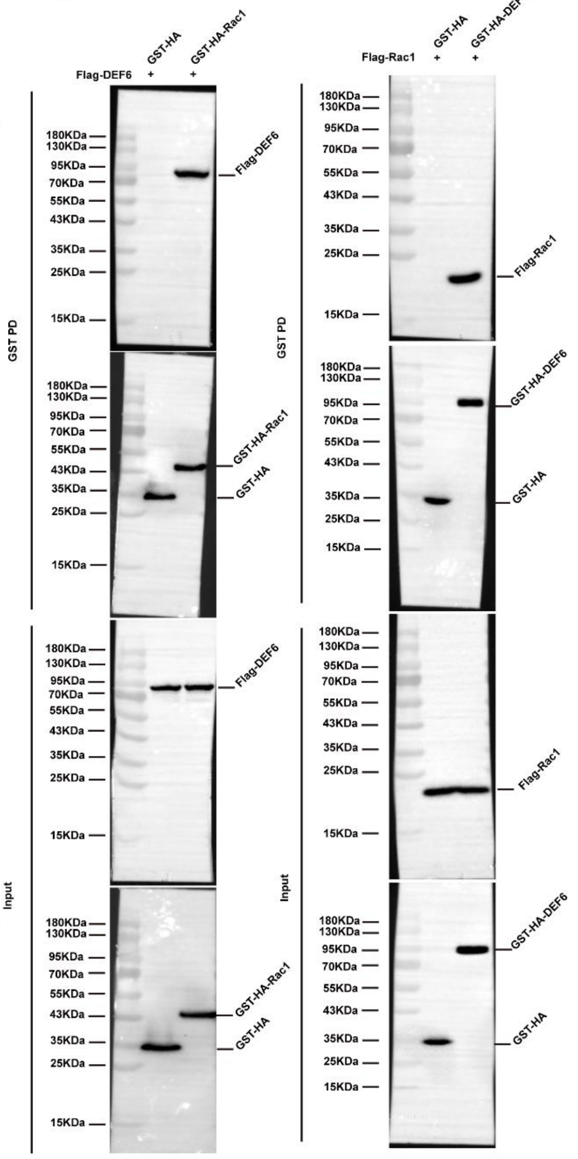
GST-HA
GST-HA-Rac1
Flag-DEF6 + +
GST PD
Input
Flag-DEF6
GST-HA-Rac1
GST-HA
GST-HA-DEF6
Flag-Rac1 + +
Flag-Rac1
180KDa
130KDa
95KDa
70KDa
55KDa
43KDa
35KDa
25KDa
15KDa

Fig6C

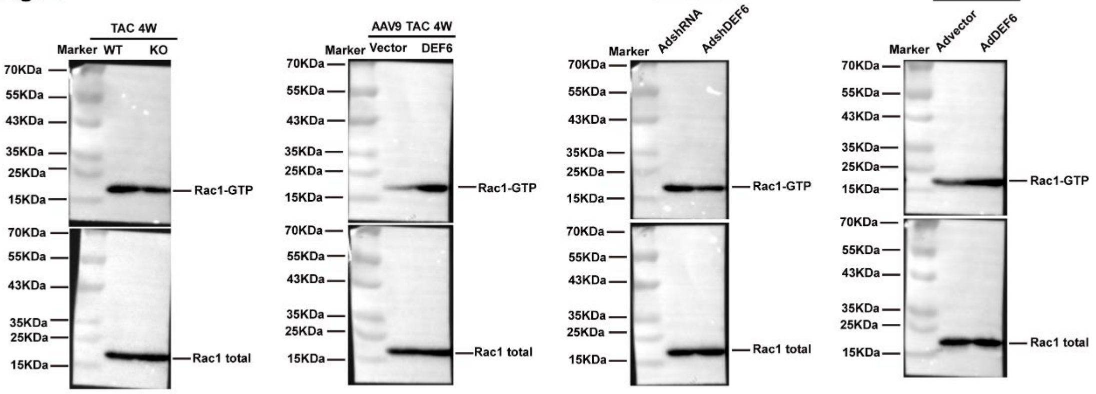

Fig6D

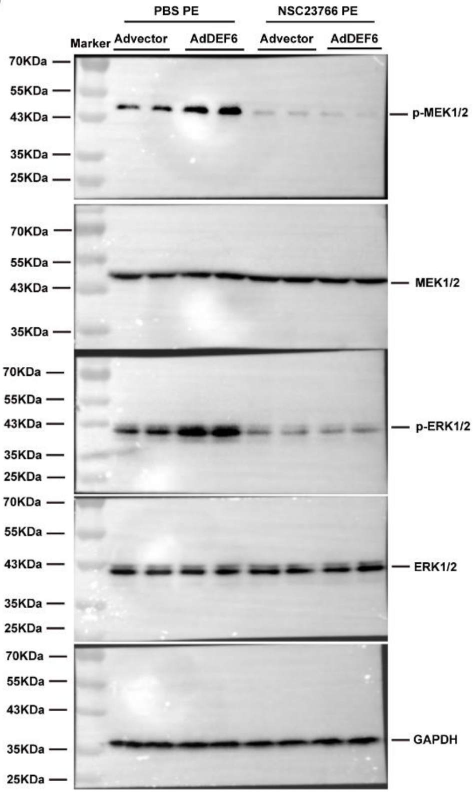

Fig6G

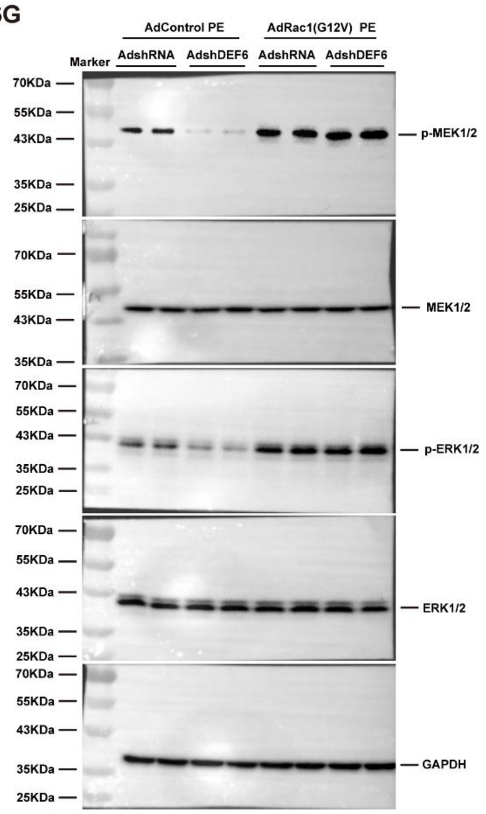

Supplement: Supplementary file 5 — Original Data File [file 41419_2023_5948_MOESM5_ESM.pdf]
